# Persistent damaged bases in DNA allow mutagenic break repair in *Escherichia coli*

**DOI:** 10.1371/journal.pgen.1006733

**Published:** 2017-07-20

**Authors:** Jessica M. Moore, Raul Correa, Susan M. Rosenberg, P. J. Hastings

**Affiliations:** 1 Department of Biochemistry and Molecular Biology, Baylor College of Medicine, Houston, Texas, United States of America; 2 Department of Molecular and Human Genetics, Baylor College of Medicine, Houston, Texas, United States of America; 3 Dan L. Duncan Comprehensive Cancer Center, Baylor College of Medicine, Houston, Texas, United States of America; 4 Department of Molecular Virology and Microbiology, Baylor College of Medicine, Houston, Texas, United States of America; A*STAR, SINGAPORE

## Abstract

Bacteria, yeast and human cancer cells possess mechanisms of mutagenesis upregulated by stress responses. Stress-inducible mutagenesis potentially accelerates adaptation, and may provide important models for mutagenesis that drives cancers, host pathogen interactions, antibiotic resistance and possibly much of evolution generally. In *Escherichia coli* repair of double-strand breaks (DSBs) becomes mutagenic, using low-fidelity DNA polymerases under the control of the SOS DNA-damage response and RpoS general stress response, which upregulate and allow the action of error-prone DNA polymerases IV (DinB), II and V to make mutations during repair. Pol IV is implied to compete with and replace high-fidelity DNA polymerases at the DSB-repair replisome, causing mutagenesis. We report that up-regulated Pol IV is not sufficient for mutagenic break repair (MBR); damaged bases in the DNA are also required, and that in starvation-stressed cells, these are caused by reactive-oxygen species (ROS). First, MBR is reduced by either ROS-scavenging agents or constitutive activation of oxidative-damage responses, both of which reduce cellular ROS levels. The ROS promote MBR other than by causing DSBs, saturating mismatch repair, oxidizing proteins, or inducing the SOS response or the general stress response. We find that ROS drive MBR through oxidized guanines (8-oxo-dG) in DNA, in that overproduction of a glycosylase that removes 8-oxo-dG from DNA prevents MBR. Further, other damaged DNA bases can substitute for 8-oxo-dG because ROS-scavenged cells resume MBR if either DNA pyrimidine dimers or alkylated bases are induced. We hypothesize that damaged bases in DNA pause the replisome and allow the critical switch from high fidelity to error-prone DNA polymerases in the DSB-repair replisome, thus allowing MBR. The data imply that in addition to the indirect stress-response controlled switch to MBR, a direct cis-acting switch to MBR occurs independently of DNA breakage, caused by ROS oxidation of DNA potentially regulated by ROS regulators.

## Author summary

Mutagenesis mechanisms upregulated by stress responses promote de novo antibiotic resistance and cross resistance in bacteria, anti-fungal-drug resistance in yeasts, and genome instability in cancer cells under hypoxic stress. Stress-induced mutagenesis is implicated as the main source of spontaneous mutagenesis that drives bacterial evolution, and may drive much of evolution generally. A widely useful model mechanism is mutagenic DNA break repair in *Escherichia coli*, in which activation of two stress responses allows error-prone DNA polymerase in the break-repair replisome and introduce misincorporations, later fixed as mutations. Both stress responses upregulate the error-prone mutagenic DNA polymerase Pol IV (DinB), suggesting that the regulation of mutagenesis to times of stress is accomplished by indirect gene upregulation, followed by DNA polymerase competition. This paper describes the discovery that the stress responses are not sufficient to allow mutagenesis caused by error-prone DNA polymerases in the break-repair replisome—damaged DNA bases must also be present—and that these are caused by reactive oxygen species in starving *E*. *coli*. We hypothesize that damaged bases may inhibit the progress of the highly processive and high fidelity replicative DNA polymerase, thus allowing the switch to error-prone DNA polymerases and mutagenesis. These findings suggest the possibility that the spontaneous mutation rate is regulated by the generation and removal of reactive oxygen, a very common byproduct of metabolism.

## Introduction

Spontaneous mutations drive development of most cancers and their resistance to therapy, aging, pathogen escape from the immune response and antibiotics, and evolution generally. Although central in all of biology and many aspects of human health, the causes of spontaneous mutagenesis have long been elusive, and generally difficult to assign with confidence. Whereas many ways to increase or induce mutagenesis are known, the origins of *spontaneous* mutations in most organisms remain speculative [1, 2].

Spontaneous mutations could potentially form by many different mechanisms [1, 2]. An early proposal [3] was that most spontaneous mutations result from spontaneous DNA damage—that repair of damaged DNA is more mutagenic than standard DNA synthesis. The mutagenicity of DNA repair has been borne out, for example, by demonstrations that repair of DNA double-strand breaks (DSBs) is mutagenic in bacteria [4–8], yeast [9], and human cancer [10–12] (reviewed [13–16]). That DNA damage underlies much of spontaneous mutagenesis was supported by discoveries that yeast antimutator mutants, i.e., mutants with lower-than-normal spontaneous mutation rate, carry mutations in DNA-damage-survival genes [17]. These genes encode alternative error-prone DNA polymerases or proteins that assist them, allowing survival of DNA damage by replication over or extension from otherwise-replication-inhibiting damaged DNA bases [18], implying that most spontaneous mutagenesis in yeast results from use of error-prone DNA polymerases during DNA-damage survival. To our knowledge, the only mechanistic detail available on spontaneous mutagenesis mechanisms is that about half of all spontaneous base-substitutions and small insertion/deletions (indels) in starving *Escherichia coli* form dependently on the proteins used in stress-inducible mutagenic DNA break repair (MBR) [7].

In MBR, alternative error-prone DNA polymerases appear to be switched into the DSB-repair replisome under the control of stress responses [6–8] (reviewed [13, 15, 16]). The central features of this mechanism have been recapitulated biochemically with purified proteins [19]. Repair of DSBs by homologous recombination requires high-fidelity replicative DNA polymerase III in unstressed cells [20]. In MBR, DSB repair uses alternative error-prone DNA polymerases, principally Pol IV (DinB) [21], but also Pols II [22] and V [7, 23], under the control of the SOS DNA-damage response and the general/starvation stress response controlled by the RpoS transcriptional activator. The sequence signature of RpoS-dependent MBR is evident across bacterial genomes [24], implicating this mechanism or similar mechanisms in most bacterial evolution. Moreover many stress-induced mutation mechanisms from bacteria to human cancer cells show similarities to *E*. *coli* MBR [13]. From these previous studies, it seemed probable that the critical step leading to MBR, and perhaps spontaneous mutagenesis generally, could be the switch from high-fidelity replicative DNA polymerases to error-prone DNA polymerases, resulting from DNA-polymerase competition promoted by upregulation of those DNA polymerases, in *E*. *coli*, by the SOS and general stress responses [13, 15, 16]. Here, we show that, for MBR, it is not sufficient to upregulate mutagenic DNA polymerases and repair DSBs; damaged DNA bases are also required.

Reactive oxygen species (ROS) are among the most common DNA-damaging agents, and are known to promote mutagenesis when in excess [25–27]. ROS attack essentially all macromolecules in cells, including DNA, RNA, proteins, and lipids [28–30]. ROS include superoxide ($\text{O}\begin{array}{c} -\\ 2 \end{array}\cdot$) and its radical derivatives, which are detoxified by superoxide dismutases upregulated in *E*. *coli* by the SoxRS oxidative stress response [31]. ROS also include hydrogen peroxide (H_2_O_2_) and its radical derivatives including hydroxyl radicals (OH^**•**^), which are detoxified by catalases and alkylhydrogenperoxidases upregulated by the *E*. *coli* OxyRS oxidative stress response [30–32]. Oxidative damage to proteins is characterized by carbonylation (reviewed by [33]), whereas ROS damage to DNA includes mainly base modifications 7,8-dihydro-8-oxo-deoxyguanosine (8-oxo-dG) and thymine glycol, in addition to other modified bases and nicks [28, 29].

The presence of 8-oxo-dG in DNA results both from incorporation of oxidized dGTP from the deoxynucleotide triphosphate (dNTP) pool and from *in situ* oxidation of guanine (G) in DNA [34]. 8-oxo-dG is often incorporated opposite to, and templates incorporation of, an adenine (A) leading to A to C and G to T transversion mutations (e.g., [35, 36]). If 8-oxo-dG is incorporated into DNA, it can persist or be excised by base excision repair (BER). Although replication bypass of template 8-oxo-dG has been shown to occur with high efficiency [35, 37], it is found that the eukaryotic replicative polymerase pol δ is transiently inhibited at 8-oxo-G [38]. Pol δ is able to extend from the A:8-oxo-dG base pair, but not from C:8-oxo-dG. A switch to an alternative polymerase, often pol λ, allows extension from C:8-oxo-dG with no substitution mutation [38]. In *E*. *coli*, of the alternative DNA polymerases, Pol IV is responsible for most MBR mutations [21], and makes base substitutions and indel mutations, mostly 1 basepair deletions in mononucleotide repeats [39].

Pol IV is reported to make mutations in the absence of induced DNA damage when overproduced [39, 40], and SOS-upregulated levels of Pol IV are required for MBR [21, 41]. Here, we report that SOS- and general-stress-response induced overproduction of Pol IV is not sufficient for Pol IV-dependent MBR. Damaged bases in the DNA must also be present. We hypothesize that damaged bases allow the switch to use of Pol IV during MBR by pausing the replicative polymerase to allow polymerase exchange in the replisome. The findings suggest that spontaneous mutation by MBR is regulated by regulation of the intracellular level of ROS.

## Results

### MBR assay in *E*. *coli*

**Fig 1:**
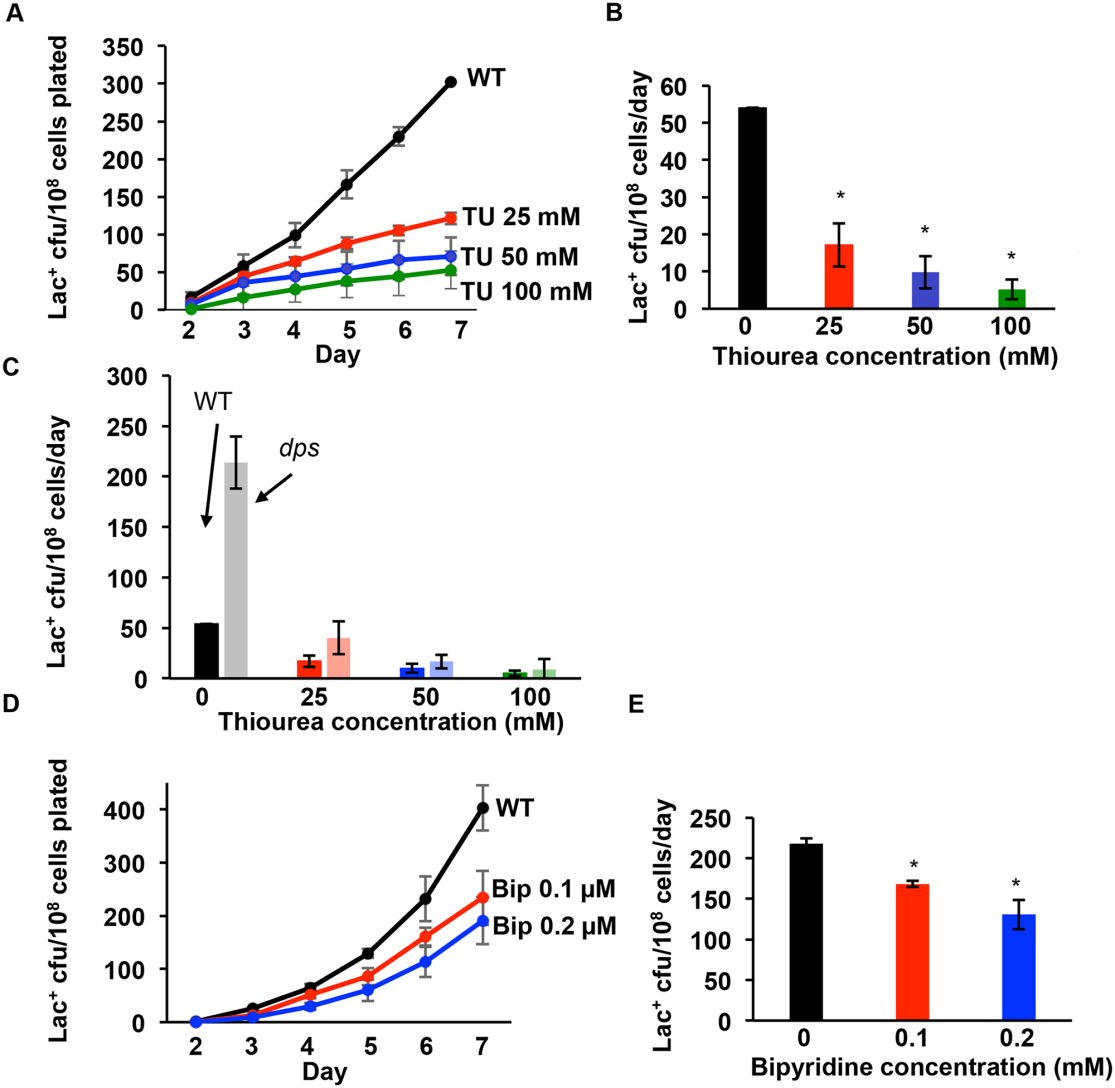
Exogenous ROS reducing agents reduce spontaneous mutagenic break repair. **(A)** Thiourea (TU) reduces Lac reversion dose-dependently. Representative experiment showing the effects of varying doses of TU on the yield of Lac^+^ mutant cfu of SMR4562. **(B)** Quantification of mutation rates from 3 experiments, days 3 to 7. Adding 25, 50, and 100 mM TU to plates reduced mutagenesis 62% ± 5.6%, 82% ± 5.6% and 90% ± 4.3% respectively (*p* = 8.3x10^-5^, 1.7x10^-5^, and 5.2x10^-5^, compared with 0mM TU, Student’s 2-tailed *t*-test). **(C)** TU eliminated the hypermutation seen in Δ*dps* strain cells dose-dependently. (**D**) 2'2-bipyridine (bip) reduces MBR dose-dependently. Representative experiment. **(E)** Either 0.1 or 0.2 μM bip reduced mutagenesis by 23% ± 4.0% and by 40% ±7.8%, respectively (*p* = 0.007 and 0.02, Student’s 2-tailed *t*-test). * indicates significantly different from the samples with zero dose of TU or bip. Strains used: wild type, SMR4562; Δ*dps*, PJH2608.

The *E*. *coli* Lac assay [42] for MBR quantifies both indels and gross chromosomal rearrangements (GCRs) as reversions of a *lacI-Z+*1bp frameshift allele in an F’ conjugative plasmid. Revertants are scored as Lac^+^ colonies formed over days of starvation on solid lactose minimal medium (e.g., Fig 1A, WT). The leaky *lac* allele reverts either by compensatory frameshift mutations (indels) or by amplification (GCRs) [43]. When placed under general/starvation-stress-response inducing conditions, or if RpoS is upregulated artificially [6,7], cells switch from faithful repair of DSBs by homologous recombination (HR) to an error-prone HR mechanism that requires the translesion DNA Pol IV, encoded by *dinB* [6, 7, 21, 44], leading to indel Lac^+^ revertants. The prevailing hypothesis for the GCR mechanism is that initial duplications of the *lacI-Z+*1bp allele are formed by a micro-homologous DSB-repair-instigated recombination event, followed by unequal crossing-over to give tandem arrays of 20 or more copies of the leaky *lac* allele [45, 46]. Both formation of GCRs and single-nucleotide alterations (SNAs, including base substitutions and indels) via MBR require DSBs [4, 6, 7, 47], HR DSB-repair proteins RecA, RuvABC [48, 49], and RecBC [4], activation of the general stress response [50, 51] and, at some genomic loci including *lac*, the RpoE (σ^E^) unfolded membrane protein response [52]. Formation of SNAs also requires the SOS response, which promotes SNA MBR by its 10-fold transcriptional upregulation of the DinB error-prone translesion polymerase [41]. SOS and DinB play no role in GCR formation [21], which instead requires DNA polymerase I [45, 53].

### Reduction of ROS through exogenous agents reduces MBR

**Fig 2:**
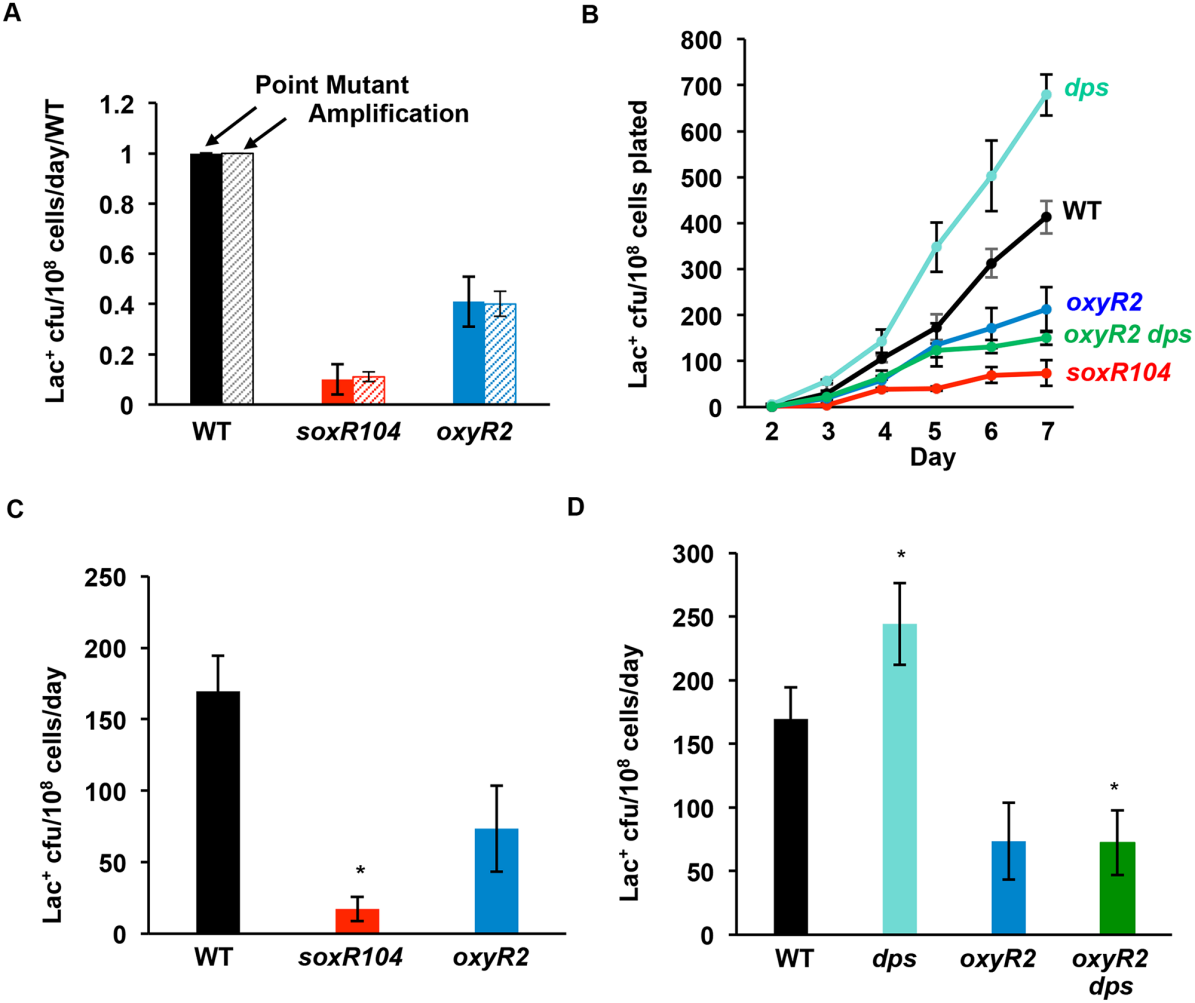
Constitutively active ROS detoxifying responses reduce MBR. (**A**) Indels (point mutations) (SNAs) and amplification (GCR), depicted here normalized to the wild-type, are reduced proportionately by *soxR104* and *oxyR2* alleles, which constitutively activate the SoxR and OxyR responses respectively. Indels (SNAs) and GCR were distinguished (Methods) in all experiments, and no disproportionate effects were detected. (**B**) Representative experiment. **(C)** Quantification of MBR rates from three experiments: *soxR104* reduced MBR by 90% ± 5.5% (*p* = 0.004, Student’s 2-tailed *t*-test). *oxyR2* reduced MBR but not quite significantly at the 5% level when tested in the wild-type (59 ± 11%), *p =* 0.07, Student’s 2-tailed *t*-test). (**D**) *oxyR2* reverses the hypermutation phenotype of Δ*dps* cells. * indicates significantly different from wild-type (*p* ≤ 0.05). Wild-type, SMR4562, black; *soxR104*, PJH2947, red; *oxyR2*, PJH3278, blue; Δ*dps*, PJH2608, pale green; *oxyR2* Δ*dps*, PJH3295, dark green. The data indicate that ROS are required for mutation formation, and that the hypermutation seen in Δ*dps* cells depends on decreased protection from ROS.

We reduced ROS levels in cells undergoing MBR using 2,2’-bipyridine (bipyridine) and thiourea (TU), chemical agents commonly used to reduce ROS levels in living cells. Both agents enter cells and prevent ROS formation (bipyridine) or quench ROS (TU) [54]. Bipyridine chelates ferrous iron and prevents it from catalyzing ROS-forming Fenton reactions [55]. TU quenches hydroxyl radicals after formation, and prevents them from damaging macromolecules [56]. We added varying amounts of bipyridine (0.1 uM and 0.2 μM) or TU (25, 50 and 100 mM) to solid minimal lactose medium on which MBR occurs, and found that Lac^+^ mutation rates were reduced dose-dependently by either ROS reducer, with a 90% ± 4.3% reduction at 100 mM TU (Fig 1A and 1B) (*p* = 5.2x10^-5^, Student’s 2-tailed *t*-test). TU also prevented the previously reported increase in MBR in cells lacking Dps [57], a stationary-phase nucleoid-compaction protein that protects DNA against ROS, again in a dose-dependent manner (Fig 1C). There is a 40% ± 7.8% reduction caused by 0.2 μM bipyridine (Fig 1D and 1E) (*p* = 0.02, Student’s 2-tailed *t*-test). Neither bipyridine nor TU affected viability of Lac^-^ mutation-reporter cells over the duration of incubation, nor did they affect the time required for formation of Lac^+^ colonies under precise reconstructions of experimental conditions (S1 Fig), indicating that mutation rate, not ability of treated cells to form colonies, was reduced by lowering ROS levels. All reductions in mutagenesis affected SNAs and amplification proportionately (an example is shown in Fig 2A). These data imply that ROS are required for MBR and suggest that Dps might inhibit mutagenesis by preventing ROS damage to DNA [57].

### Production of ROS-detoxifying enzymes reduces MBR

We reduced ROS levels in cells undergoing MBR by constitutive overexpression of either of two *E*. *coli* oxidative stress responses: the SoxRS response and the OxyRS response [31, 32]. Whereas each regulon responds to the presence of ROS in the cell, they employ different enzymes that detoxify different radical species. The SoxR response upregulates superoxide dismutases that inactivate superoxides and their derivatives [31], and the OxyR response upregulates catalases and alkylhydroperoxidases that inactivate hydrogen peroxide and its radical derivatives including hydroxyl radicals [32]. We used the *oxyR2* [58] and *soxR104* [59] alleles (separately), which cause constitutive expression of each response at induced levels, in the absence of oxidative inducers, to reduce ROS levels normally present during MBR. *soxR104* reduced Lac^+^ MBR mutation rate, conferring a 90 ± 5.5% reduction (Fig 2B and 2C) (*p =* 0.004, Student’s 2-tailed *t*-test). Depression of MBR by *oxyR2* (59 ± 11%) was not quite significant at the 5% level (*p* = 0.07, Student’s 2-tailed *t*-test) when tested in the wild-type strain. However, *oxyR2* completely eliminated the Δ*dps-*mediated increase in Lac^+^ MBR, showing a 70 ± 7.8% reduction in mutation frequency (Fig 2B and 2D), (*p* = 0.014, Student’s 2-tailed *t*-test) to the same level as the strain with *oxyR2* alone (*p* = 0.98, Student’s 2-tailed *t*-test). The data indicate that ROS are required for MBR generally, and we infer that the mechanism of Dps inhibition of MBR is its protection of DNA against ROS.

### ROS are required for stress-induced mutagenesis

**Fig 3:**
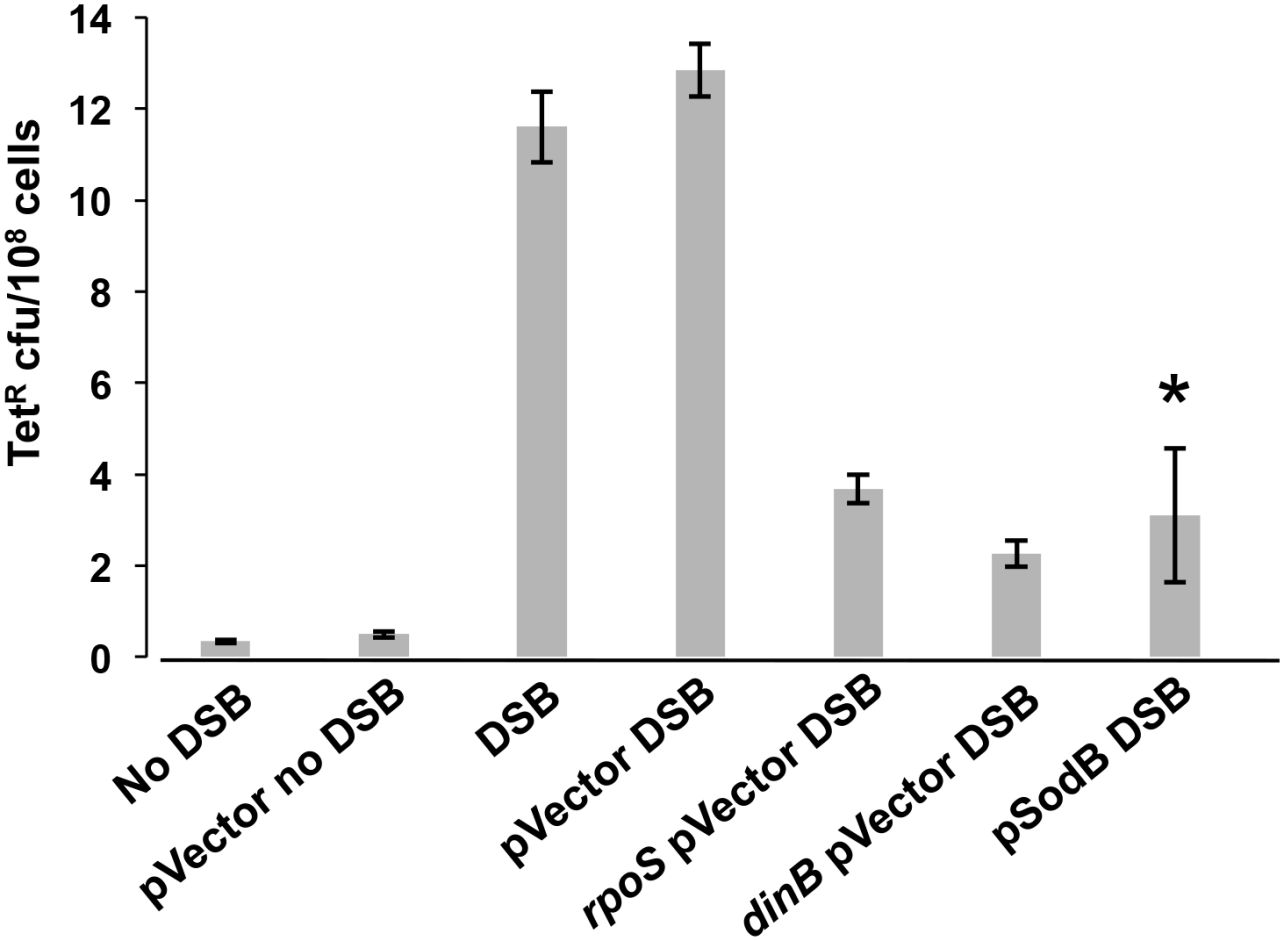
DSBs do not substitute for ROS in MBR. In the Tet MBR assay, DSBs are induced in starved cells by induction of I-*Sce*I double-strand endonuclease cleavage at an I-*Sce*I cutsite near the chromosomal *tet* indel mutation-reporter gene [6, 7]. SodB overexpression is induced by IPTG, and reduces TetR mutant frequency even when DSBs are provided by I-SceI. Thus, I-*Sce*I cannot substitute for ROS, and ROS promote MBR other than or in addition to by causing DSBs. * significantly different from the isogenic empty-vector control (pVector) at the 5% level. Δ*rpoS* and *dinB50* positive-control strains with empty vector and DSB induction demonstrate RpoS- and DinB-dependent MBR, here and in Fig 5. Strains used: no DSB, SMR10798; no DSB pVector, PJH3237; DSB, SMR10866; DSB pVector, PJH3232; DSB Δ*rpoS* pVector, PJH3240; DSB *dinB50* pVector, PJH3231; DSB p*SodB*, PJH3257.

We removed ROS from cells by overexpressing *sodB*, which encodes a superoxide dismutase. We used a plasmid from the mobile plasmid library [60] that carries each gene under the control of an isopropyl β-D-1-thiogalactopyranoside (IPTG)-inducible promoter in cells used for the Tet MBR assay [7]. The Tet MBR assay reports on indel mutations that revert a chromosomal *tetA* +1bp frameshift allele during starvation in liquid minimal medium. In the Tet assay, DSBs are induced near a chromosomal *tet* mutation-reporter gene by the I-*Sce*I site-specific double-strand endonuclease expressed in F-plasmid-free cells during starvation. *tet* reversion in this assay requires the key MBR proteins, as in Lac^+^ MBR, including RpoS, the SOS response regulators, Pol IV, and DSB-repair proteins [6, 7, 16]. The Tet assay differs from the Lac MBR assay not only in having no F-plasmids present, but also in having no selection for the reverted allele during the starvation stress; Tet-resistant (Tet^R^) revertants are selected as Tet^R^ cfu after the cells are rescued from starvation in liquid. The Tet assay does not measure GCR. We found that over-expression of *sodB* reduced Tet^R^ MBR mutant frequencies 76% ± 12%, (Fig 3, 0.003, Student’s 2-tailed *t*-test). These data show, by enzymatic removal of ROS, that ROS are required for starvation-stress-induced MBR. We conclude that MBR requires ROS for mutagenesis.

### ROS promote MBR other than by formation of DSBs

We eliminated several possible mechanisms for the requirement for ROS in MBR. Repair of oxidized DNA bases might lead to formation of spontaneous DSBs that instigate MBR [4, 6, 7, 47]. Base excision repair proceeds by excision of the damaged base by specific DNA glycosylases forming an abasic (AP) site, followed by nicking at that site by an AP endonuclease. If not repaired, the nick could produce a one-ended DSB when replicated, via replication fork breakage [61]. Using the Tet MBR assay, we find that even in the presence of an I-*Sce*I-endonuclease-generated DSB, over-expression of *sodB* still reduced Tet^R^ MBR as described above (Fig 3). We show that induction of DSBs by I-*Sce*I is effective when SodB is overexpressed (S2 Fig). We conclude that ROS promote MBR other than or in addition to by promoting spontaneous DSBs.

### ROS do not promote MBR via oxidation of proteins

**Fig 4:**
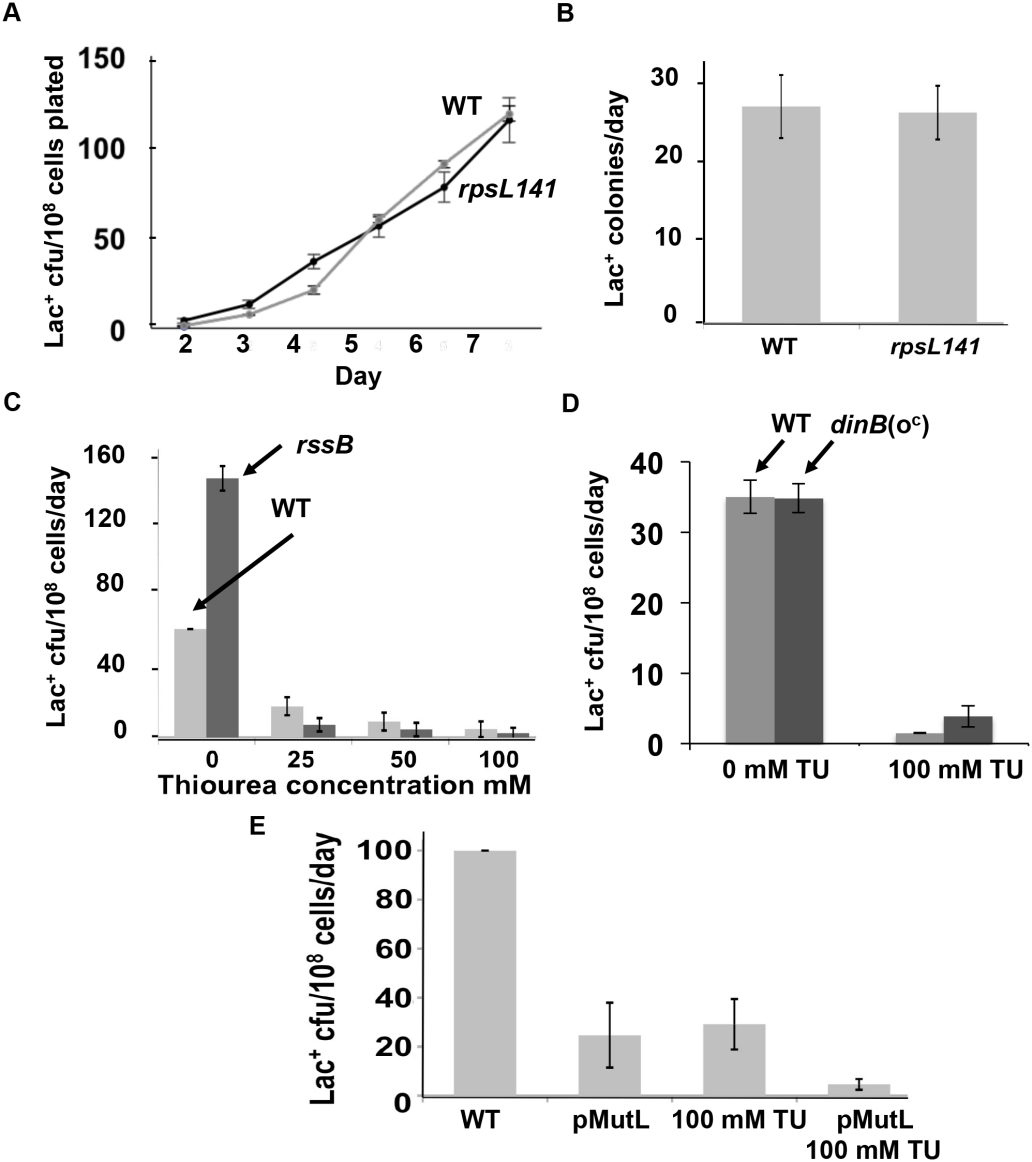
ROS promote MBR other than or in addition to by upregulation of the RpoS or SOS responses, oxidation of proteins, or saturation of mismatch repair. (**A, B**) The hyper-accurate ribosomal protein allele *rpsL141* [62] does not reduce MBR, implying that ROS promote MBR other than by producing carbonylated proteins, which are reduced by this allele. **(A)** Representative experiment. **(B)** Mean ± SEM of three experiments. **(C)** Artificial upregulation of RpoS levels in Δ*rssB* strains does not restore MBR in TU-treated cells, implying that ROS promote MBR other than or in addition to by upregulation of RpoS. **(D)** An operator-constitutive *dinB*(o^c^) allele, which substitutes for the SOS response in MBR [41], does not substitute for depletion of ROS by TU. (**E**) Over-expression of *mutL*, the limiting mismatch repair (MMR) component in MBR [63], causes additive reduction of MBR with TU treatment, implying that TU and MMR reduce MBR by different pathways, indicating that ROS promote MBR other than by saturation of MMR capacity. Strains used: wild type (WT) SMR4562; *rpsL141*, PJH3178 Δ*rssB*, SMR12566; *dinB*(o^c^) SMR10308; P_*BAD*_*MutL*, SMR15378. P_*BAD*_*MutL* was derepressed by absence of glucose in the medium.

ROS damage proteins, lipids, RNA and DNA [30]. Oxidation of proteins, which causes carbonyl groups, can inhibit protein function (reviewed by [33]). We used a mutation that reduces cellular levels of carbonylated proteins: *rpsL141*, which encodes a hyper-accurate ribosomal protein that reduces intracellular carbonylated protein content [62]. We found that Lac assay MBR rates were unaffected by *rpsL141* (Fig 4A and 4B), implying that promotion of MBR by ROS is not mediated by oxidation of proteins.

### ROS promote MBR other than via induction of the general or SOS stress responses

We excluded the possibility that ROS promote MBR by inducing the RpoS response by assaying MBR in an *rssB* mutant in the presence of TU. RssB targets RpoS, the transcriptional activator of the general stress response, for degradation by the ClpXP protease, so that when *rssB* is deleted, RpoS levels increase, artificially upregulating the RpoS response [64]. If ROS promoted MBR solely by inducing the general stress response, Δ*rssB* would be expected to counter the anti-MBR effect of TU treatment. We find that TU treatment reduced MBR dose-dependently in Δ*rssB* cells as in wild-type cells (Fig 4C), (*p* = 0.16; 0.92; 0.95, comparing WT with *rssB* at 25, 50, and 100 mM TU, respectively; Student’s 2-tailed *t*-test). We conclude that ROS probably promote MBR other than or in addition to by activation of the general stress response.

The SOS DNA-damage response promotes MBR via its 10-fold transcriptional upregulation of Pol IV error-prone DNA polymerase, and is not needed for MBR in cells with an operator-constitutive (constitutively over-expressing) *dinB* allele [41]. We find that the *dinB*(o^c^) allele did not counter TU treatment [Fig 4D, *p* = 0.001 for wild-type (WT) versus WT +100mM TU; *p* = 0.0003 comparing *dinB*(o^c^) with *dinB*(o^c^) +100mM TU, Student’s 2-tailed *t*-test]. By contrast wild-type and the *dinB*(o^c^) strain are not different [*p* = 0.95; and *p* = 0.10 for WT with TU *versus dinB*(o^c^) with TU, Student’s 2-tailed *t*-test], implying that ROS promote MBR other than or in addition to by activation of the SOS response.

### Oxidative damage promotes MBR other than by titration of mismatch repair

We tested the possibility that excessive base damage from ROS might overwhelm mismatch repair, making it unable to correct base misincorporations by DinB. MutL, a required component of mismatch repair, was shown to become limiting during MBR [63] and for polymerase errors generated by a hyper-mutator Pol III [65]. We combined TU treatments with overproduction of MutL, to test whether oxidized DNA bases saturate mismatch repair capacity during MBR. Previously, increasing mismatch repair capacity by overproducing MutL reduced Lac^+^ MBR approximately 4-fold [63]. If ROS promoted MBR by saturating mismatch repair capacity, MutL overproduction and TU inhibition of MBR would be expected have an epistatic relationship, implying their action in the same pathway. By contrast, we found that 100mM TU combined with MutL overproduction reduced Lac^+^ MBR additively (Fig 4E), *P* = 0.005, 0.002, 0.838, and 0.035 for WT compared with MutL overproduction; WT compared with TU; WT + TU compared with MutL overproduction; and WT + TU compared with MutL overproduction + TU, respectively, Student’s 2-tailed *t*-test. The data imply that ROS promote MBR other than by causing saturation of mismatch repair.

### ROS promote MBR via persistent oxidized guanine in DNA

**Fig 5:**
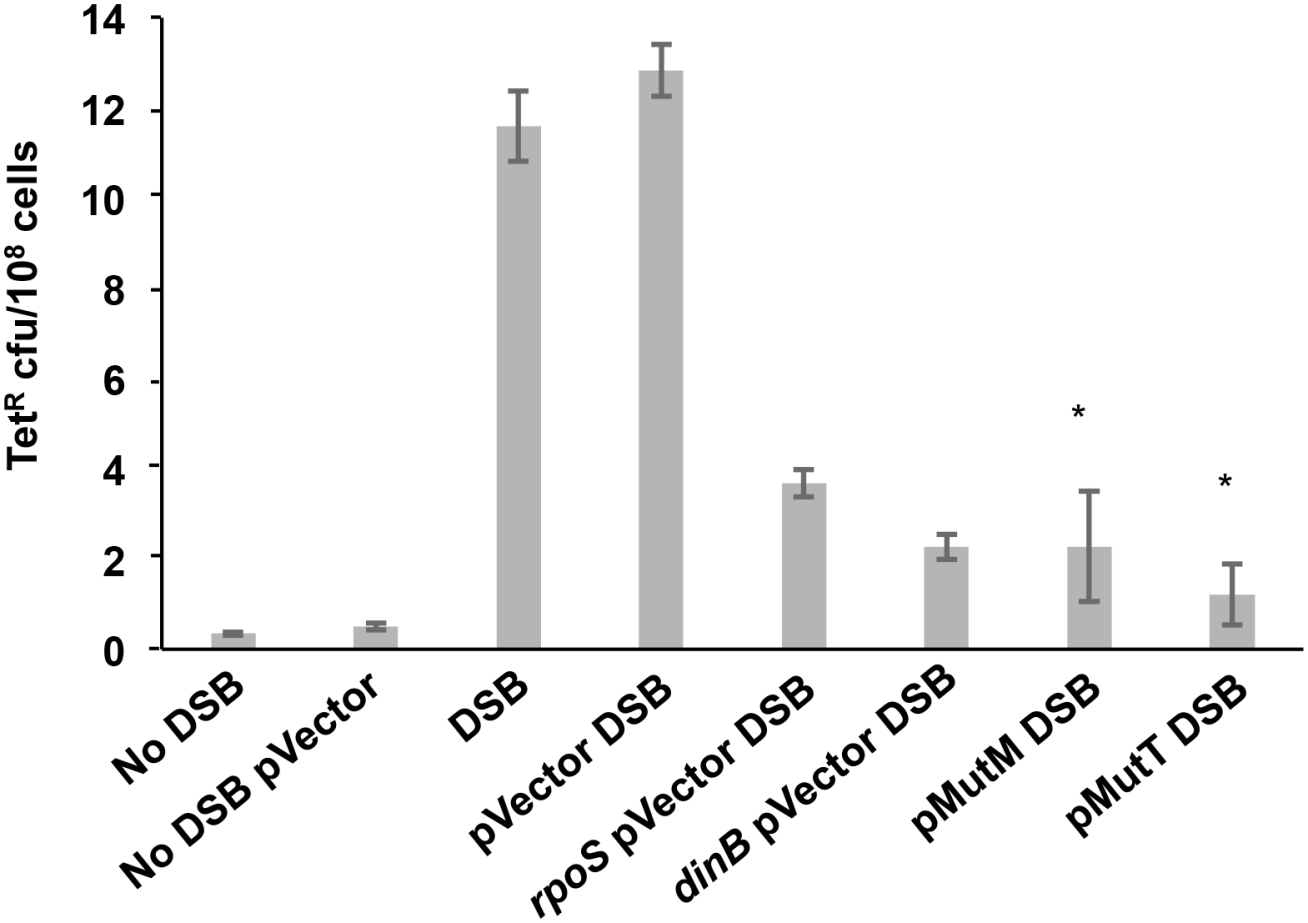
Persistent oxidized guanine in DNA is required for MBR. Overproduction of MutM 8-oxo-dG DNA glycosylase and MutT 8-oxo-GTP diphosphatase nucleotide triphosphate pool sanitizer reduces MBR in the Tet assay. Overproduction of MutM and MutT reduced Tet^R^ mutant frequency 82% ± 9.7% and 91% ± 5.3%, respectively compared with the vector control (*p* = 0.0015 and 0.0002, Student’s 2-tailed *t*-test). The data indicate, first, that 8-oxo-dG in DNA is required for MBR, second, that the effects of 8-oxo-dG on MBR result mainly from incorporation of 8-oxo-dG from the dNTP pool, and third, that 8-oxo-dG must remain in DNA for MBR to occur, because if 8-oxo-dG is removed by the MutM DNA glycosylase, MBR is reduced. * significantly different from the empty vector control (pVector) at the 5% level. Strains used: No DSB, SMR10798; no DSB pVector, PJH3237; DSB, SMR10866; DSB pVector, PJH3232; DSB *rpoS* pVector PJH3240; DSB *dinB50* pVector PJH3231; DSB p*MutM*, PJH3233; DSB p*MutT*, PJH3256.

We found that removing oxidized guanine from DNA reduces MBR. *E*. *coli* has three proteins that specifically reduce the mutagenic effects of 8-oxo-dG. MutM is a DNA glycosylase that excises 8-oxo-dG from DNA, MutY is another glycosylase that excises mispaired adenine opposite to G or 8-oxo-dG, and MutT hydrolyses 8-oxo-dGTP in the nucleotide triphosphate pool, reducing incorporation of 8-oxo-dG into DNA. We (separately) overproduced MutM, MutT, and MutY in Tet MBR assay cells using mobile plasmid library plasmids [60], and measured Tet^R^ mutant frequencies. We detected no decrease in cell viability during these experiments or in growth of strains containing these plasmids induced with 1mM IPTG (S3 Fig). We found that overproduction of MutM and MutT reduced Tet^R^ MBR 82% ± 9.7% and 91% ± 5.3%, respectively, compared with the vector-only control (*p* = 0.0015 and 0.0002, Student’s 2-tailed *t*-test) (Fig 5). Δ*rpoS* and *dinB* positive-control strains confirm that mutagenesis is via the canonical MBR pathway. In eight repeated experiments we observed a small, but not significant, decrease in Tet MBR with MutY overproduction (32% ± 9.5% decrease, *p* = 0.15, Student’s 2-tailed *t*-test), suggesting that mispaired adenines have, at most, a minor effect on promotion of MBR. We conclude that persistent unrepaired 8-oxo-dG in DNA is required for MBR, and that ROS promote MBR *via* the presence of 8-oxo-dG in DNA. Furthermore, because the increased removal of 8-oxo-dG from DNA eliminates most mutagenesis, we conclude that whereas oxidized proteins or lipids, or oxidative lesions in RNA might play a role in MBR, it is at most a small one. The strong effect of overproduction of MutT, the 8-oxo-dGTP diphosphatase, implies that the MBR-promoting 8-oxo-dG in DNA results mainly from incorporation from the nucleotide pool rather than *in situ* oxidation of DNA.

### Other base damage in DNA can substitute for 8-oxo-dG in MBR

**Fig 6:**
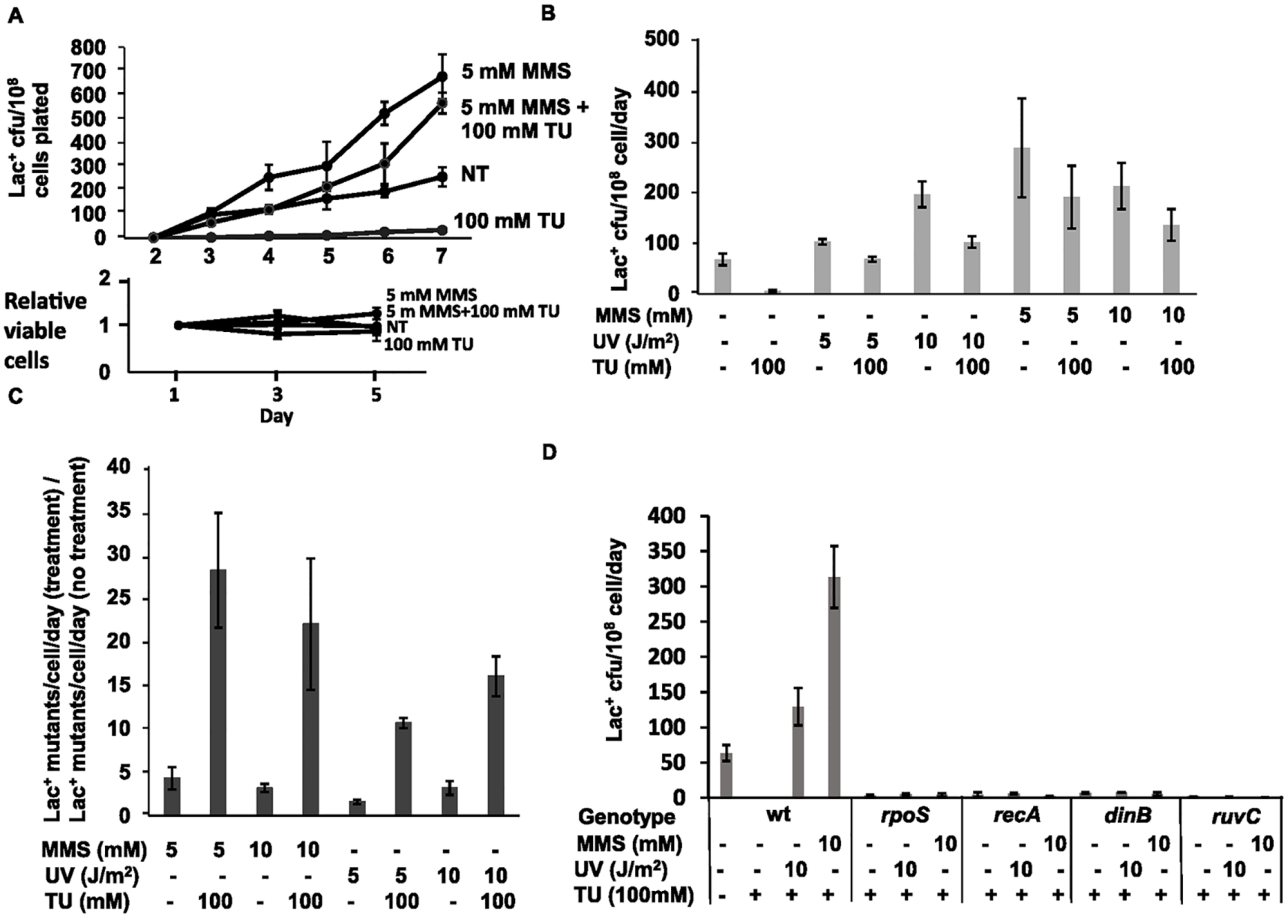
Base-damage treatments substitute for ROS-induced 8-oxo-dG in MBR. **(A)** Methyl methanesulphonate (MMS) or UV-C light restore mutagenesis to ROS-scavenged cells. Representative experiment. ROS are reduced with 100 mM thiourea (TU); NT, no treatment. Below is a representative example of viability data showing that TU treatment and MMS/UV pulse treatment do not affect tester-cell viability during the mutagenesis assay. **(B)** Quantification from multiple experiments. Non-lethal doses of MMS (5 mM and 10 mM) or ultraviolet C light (UV) (5 and 10 J/m^2^) restored mutagenesis to cells treated with high levels of TU. For 5 J/m^2^ UV, there is an 11 ± 0.6-fold increase (*p* = 0.0004, Student’s 2-tailed *t*-test), compared with TU treatment with no mutagen. For 10 J/m^2^ UV, the increase is 16 ± 2.3-fold (*p* = 0.001, Student’s 2-tailed *t*-test; for 5mM MMS, 28 ± 6.6-fold (*p* = 0.049, Student’s 2-tailed *t*-test) and for 10mM MMS, 22 ± 7.8-fold (*p* = 0.02, Student’s 2-tailed t-test). **(C)** MMS and UV treatments have much stronger effects on Lac^+^ mutant formation when ROS are scavenged by TU than when they are not. Although both treatments increased basal Lac^+^ mutation rates, 3 ± 0.5-fold with 10 mM MMS and 3.0 ± 0.8-fold with 10 J/m^2^ UV, the increases caused by MMS or UV were much greater in TU treated than untreated cells, indicating that MMS and UV treatments specifically substitute for the requirement for ROS (*p* = 0.006 for both, Student’s 2-tailed *t*-tests, when comparing mutagen +TU/NT+TU with mutagen only/NT with no mutagen). Data are from Fig 6B. **(D)** The mutagenesis restored to ROS-depleted cells by UV and MMS is on-pathway MBR, requiring RpoS, RecA, DinB and RuvC. Thus, the requirement for damaged bases in DNA for MBR is not specific to 8-oxo-dG, but can be provided by other replication-hindering DNA damage. Strains used: Wild-type (WT), SMR4562; Δ*rpoS*, PJH1399; *recA*, SMR4610; *dinB50*, SMR5889; *ruvC*, SMR6906.

We tested the hypothesis the 8-oxo-dG in DNA might promote MBR by pausing the high-fidelity replicative DNA Pol III, active in DSB repair [20], which might allow Pol IV and other alternative DNA polymerases to switch into the active position in the repair replisome. If this were the case, then 8-oxo-dG would not be required as a specific intermediate in MBR; any fork-stalling DNA-base damage traversed or extended from by translesion DNA polymerases would be expected to substitute for 8-oxo-dG. We tested whether other persistent DNA damage could bypass the requirement for ROS and 8-oxo-dG in MBR by using methyl methanesulfonate (MMS), which methylates DNA, forming mainly *N*^7^-methyldeoxyguanosine and *N*^3^-methyldeoxyadenosine [66], and ultraviolet-C light (UV-C), which creates pyrimidine dimers and 6–4 photoproducts (reviewed by [67]), all of which stall the replicative DNA Pol III and can be traversed by translesion DNA polymerases [68–70]. We pulse-treated starved cells with nonlethal doses of MMS or UV-C before plating on minimal lactose medium with 100mM TU to scavenge ROS. Whereas both MMS and UV-C increased basal mutation rates slightly (3-fold for each treatment, Fig 6A and 6B), the increase in mutagenesis seen with TU + MMS or UV treatments compared with TU alone was many-fold greater (Fig 6C), 22 ± 7.8-fold for 10mM MMS (*p* = 0.02, Student’s 2-tailed t-test), and the increase is 16 ± 2.3-fold for 10 J/m^2^ UV, (*p* = 0.001, Student’s 2-tailed t-test). The data show a robust return to mutagenesis caused by those base-damaging agents after removal of ROS. The data imply that other base damages can substitute for 8-oxo-dG in mutagenesis. MMS or UV treatment completely countered the effect of 100 mM TU (Fig 6B). Further, we show that the mutagenesis restored by MMS or UV in the absence of ROS is “on-pathway” MBR, in that mutagenesis depends completely on RecA, RpoS, Pol IV, and RuvC (Fig 6D). These data show that MMS and UV did not activate an alternative mutagenesis mechanism(s), but rather restored MBR to ROS-depleted TU-treated cells.

Both MMS and UV-C might induce oxidative damage in addition to their more common lesions. UV-C can induce 8-oxo-dG formation in HeLa cells and in DNA, although 8-oxo-dG induction was about 2000-fold lower than pyrimidine dimer induction [71]. MMS induces ROS in yeast [72], however, this requires the presence of glucose in the medium [73, 74], and glucose is absent from the medium of starved *E*. *coli* cells, making this unlikely. In Fig 1D we used a high dose (100mM) of TU to scavenge ROS and found that the four-fold increase in MBR caused by Δ*dps* is completely blocked by 100mM TU. Given the expected much greater levels of ROS in Δ*dps* than WT cells and the ability of TU to quench the ROS in Δ*dps* cells, these data suggest that there is still substantial ROS quenching capacity when TU is applied to wild-type cells. All of these data imply that MMS and UV-C promoted MBR not by promoting 8-oxo-dG in DNA, but rather by their abundant standard base damages. The slightly lower mutagenesis seen in the presence of TU for all mutagen treatments in Fig 6B (*p* = 0.01, 0.03, 0.40, and 0.20, Student’s 2 tailed *t*-test for 5 J/m^2^ UV, 10 J/m^2^ UV, 5 mM MMS, and 10 mM MMS, respectively, in the presence versus the absence of TU) might reflect a low level of MBR from oxidative damage when UV and MMS are used. However, most MBR in MMS or UV treated cells arises from non-oxidative base damage, which is not prevented by TU. These data demonstrate that persistent damaged bases in DNA are needed generally for MBR, and imply that the DNA substrate for MBR is more than a simple DSB undergoing HR repair: that, additionally, damaged DNA bases must be present in the DNA for MBR to occur.

## Discussion

Discovered in *E*. *coli*, MBR is an important model molecular mechanism of mutagenesis later discovered in other bacteria, yeasts, flies, and human cells and cancers (reviewed [13]). Until this report, the *E*. *coli* MBR mechanism was defined by its requirements for various *trans*-acting components, but only a single central *cis*-acting DNA component: DNA DSBs, which are required for spontaneous MBR [4, 6, 7, 47] and at which mutagenesis is focused [6, 8]. The *trans*-acting proteins are those of DSB repair, low-fidelity translesion DNA polymerases, transiently insufficient mismatch repair and stress-response regulators (reviewed [13]), including a large gene network, in which most act upstream of the stress-response regulators in stress sensing and signal transduction that activates the RpoS, SOS and membrane stress responses [75]. Supporting this binary view, although transcriptional R-loop RNA/DNA hybrids and the Mfd RNA polymerase translocase are also required for MBR [47], they and the membrane stress response [45] promote MBR by promoting spontaneous DSBs at some loci, and are not needed when endonuclease-generated DSBs are provided [47, 52]. By contrast, the data presented here show that a separate, additional and unrelated DNA substrate and event must occur for MBR: damaged DNA bases. We found that spontaneously, during starvation-induced MBR, ROS-promoted 8-oxo-dG in DNA underlies most MBR (Figs 1–3 and 5), and that 8-oxo-dG does not promote MBR solely by generation of DSBs (Figs 3 and 5), and so is a distinct, new *cis*-acting DNA component of the MBR molecular mechanism. This mechanism applies both to SNA and GCR MBR mechanisms (Fig 2A). Other roles for ROS in stress response activation, protein oxidation, and mismatch repair saturation were shown not to underlie the ROS role in MBR (Fig 4). Because the persistent 8-oxo-dG in DNA that drives spontaneous MBR during starvation can be substituted by other generic damaged bases caused by UV light or the alkylating agent MMS (Fig 6), the data indicate that damaged bases generally are required for MBR, and in a role separable from specifically oxidized guanine.

Whereas previously we found that the stationary-phase upregulated ROS-detoxifying protein Dps inhibits MBR [57], here we find that the Dps inhibition of MBR is consistent with its effects on ROS reduction (Figs 1C and 2D).

### Model: Damaged bases promote DNA polymerase switching to license MBR—SNAs

**Fig 7:**
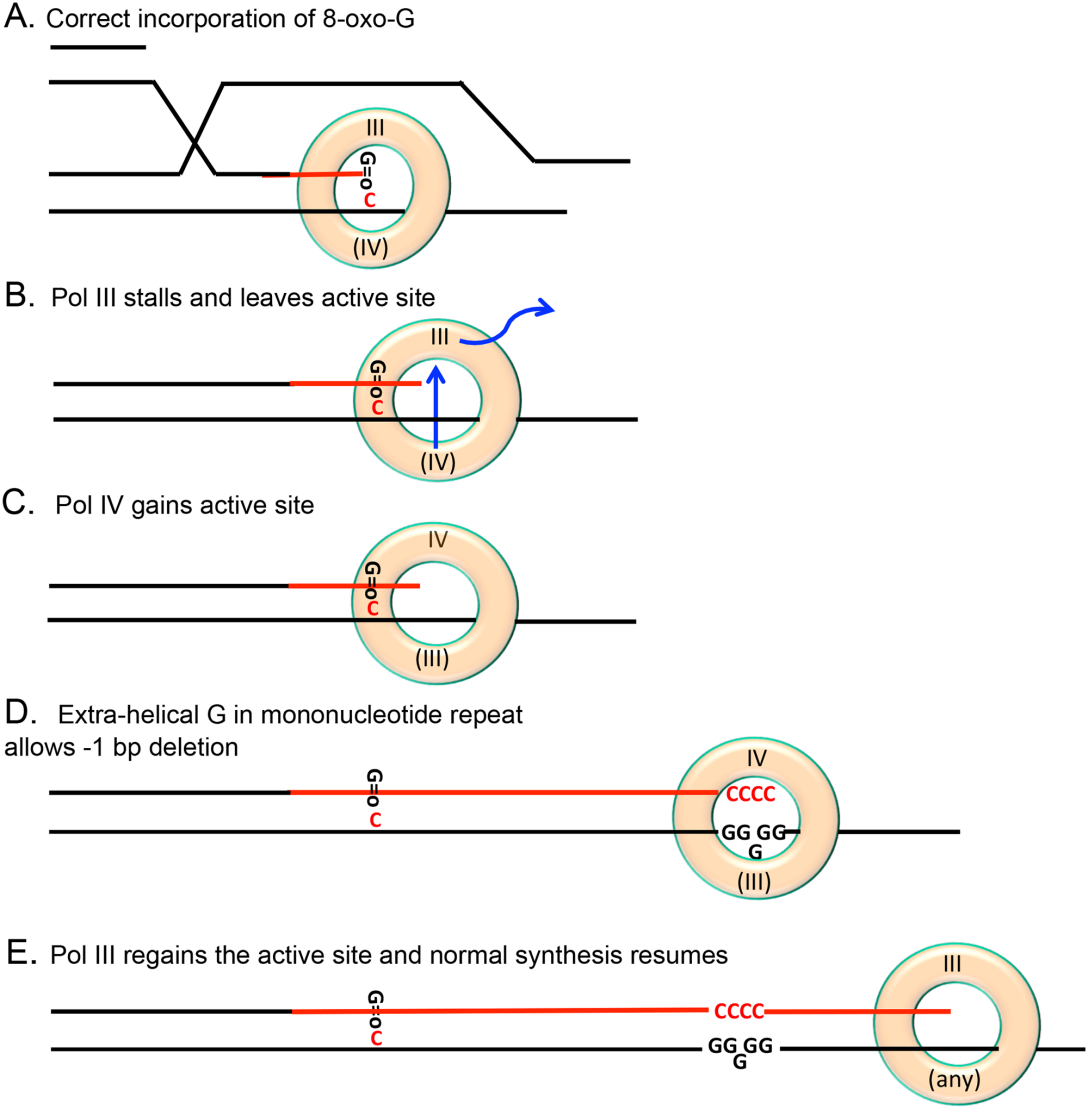
Model: Replisome pausing at damaged bases allows DNA polymerase switching and MBR: Example of oxidized bases allowing Pol IV-dependent indels at non-damaged sites downstream. This example illustrates a general model in which the switch from the processive, high-fidelity DNA Pol III used in DSB repair [20] to any of the alternative mutagenic DNA polymerases results from Pol III pausing at a damaged base, either in the template or newly added to the 3’-primer end. One way that 8-oxo-dG could allow Pol IV-mediated MBR mutations (at other DNA bases) is shown here. (**A**) A D-loop made during DSB-repair of a one-ended DSB has invaded a sister DNA molecule (parallel lines, base-paired DNA) and primed new synthesis (red line) using Pol III. Pol III inserts 8-oxo-dG, here opposite a template C [38], or could stop after inserting C at 8-oxo-dG in the template (not illustrated). (**B**) Pol III pauses because it extends poorly from an 8-oxo-dG primer end paired with template C [38]. We suggest that the pausing allows Pol III to leave the active position on the β-clamp, and an alternative DNA polymerase on the β-clamp (Pol IV/DinB shown here) to move into the β-clamp active position. (**C**) Pol IV in the active position extends synthesis from the 8-oxo-dG primer opposite template C (or, not illustrated, bypasses an 8-oxo-dG in the template strand, inserting the correct base or an incorrect base), and continues synthesis making indel (and/or base substitution) errors at mononucleotide repeat sequences, at which it is most error-prone. (**D**) Eventually Pol IV pauses because of its limited processivity [39], allowing Pol III to resume. Black lines represent pre-existing nucleotide chains and red lines represent new synthesis. The β-clamp is represented as an orange doughnut. The β-clamp site with the active DNA polymerase is illustrated at the top of the β-clamp, and the inactive DNA Pol binding site at the bottom in parenthesis. Blue arrows in (B) indicate movements of polymerases on and off the β-clamp. G = o indicates 8-oxo-dG.

We suggest a model in which the role of damaged bases in MBR is to allow the switch from high-fidelity DNA Pol III [20] to low-fidelity translesion DNA polymerases during homologous recombinational DSB repair, thus allowing MBR. A requirement for Pol III pausing to allow a switch to Pol IV has been shown previously [76–78]. A simple example of this idea is illustrated in Fig 7 for the formation of SNAs. The general model is that replication stalling at base damages that are not easily replicated or extended from by Pol III allows switching to alternative DNA polymerases, some of which are mutagenic, by pausing the replisome so that the DNA polymerases can switch.

Damaged bases can be repaired in double-stranded DNA, but in single-stranded DNA at the replication fork they must be bypassed by translesion synthesis (TLS) [79]. TLS often uses alternative DNA polymerases to synthesize across damaged bases or to extend from the resultant mismatches after incorporation [80]. TLS polymerases include the Y-family polymerases Pol IV/DinB and Pol V in *E*. *coli*. DNA polymerases in the replisome and in repair complexes are attached to the β processivity clamp, which is the structural homolog of proliferating cell nuclear antigen (PCNA) in yeast and mammalian cells [81]. In both *E*. *coli* and eukaryotes, replicative polymerases might pause when they encounter a damaged base, either before incorporation or before extension, allowing a TLS polymerase that is already loaded on the β-clamp [77] to move onto the DNA primer end and synthesize past the damaged base or to extend beyond it. In the version of this general model shown in Fig 7, we show Pol IV (DinB) replacing Pol III in the active position on the beta clamp (Fig 7B). The replicative Pol III can incorporate 8-oxo-dG opposite A (not illustrated) or C (Fig 7A) [38]. Assuming that findings about eukaryotic polymerases [38] can be applied to *E*. *coli*, Pol III would then pause transiently because of poor ability to extend synthesis from the 8-oxo-dG paired with template C [38]. Alternatively Pol III could pause at an 8-oxo-dG in the template strand paired with C on the primer (not illustrated) [38]. In these cases, we suggest that stalling of Pol III allows it to be shifted out of the β processivity clamp site carrying the active DNA polymerase (top of β-clamp doughnut shown Fig 7), and a DNA polymerase in the clamp inactive position (bottom of β-clamp, Fig 7) switched into the active position (Pol IV shown switching places with Pol III, Fig 7B and 7C). With Pol IV in the β-clamp active DNA polymerase position, synthesis can resume from the 8-oxo-dG primer opposite template C (red line, Fig 7C) or from C across from 8-oxo-dG in the template (not illustrated), and base-substitution and/or indel mutations can be made downstream after the switch, for example when Pol IV encounters a run of Gs (Fig 7D and 7E), a slippery template sequence at which Pol IV makes errors with high probability [72]. Pol IV is processive for about 400 nucleotides [39]. Pol IV and its orthologous Y-family DNA polymerases in other species including human can make indel mutations because their active sites can accommodate extrahelical bases in mononucleotide repeats in the template strand, and thus insert fewer nucleotides than are on the template [82, 83]. Their most common errors are base substitutions, however. The data presented here reveal that the availability of alternative DNA polymerases is not sufficient to allow mutagenesis, which requires polymerase switch, supporting models like this one.

### Alternative DNA polymerases in MBR

All three DNA damage-response (SOS)-induced polymerases, Pols II, IV and V, contribute to stress-induced MBR SNA formation [7, 21–23]. Pol IV promotes 85% of indels in Lac MBR, and can increase mutation rates over a thousand-fold in the absence of exogenously induced DNA damage when overproduced in *E*. *coli* [40]. The remaining 15% of Lac MBR indels require Pol II [22]. Pol V promotes MBR base substitutions [23] and some indels [7]. GCR, seen as amplifications, does not require any of the SOS-inducible DNA polymerases, but requires DNA Pol I [45]. Thus all stress-induced MBR requires alternative DNA polymerases. Presumably the relative availability of the different DNA polymerases will influence which polymerases are switched into the MBR replisome and replace Pol III at the active position on the beta clamp.

### Model: Damaged bases promote DNA polymerase switching to license MBR—GCRs

**Fig 8:**
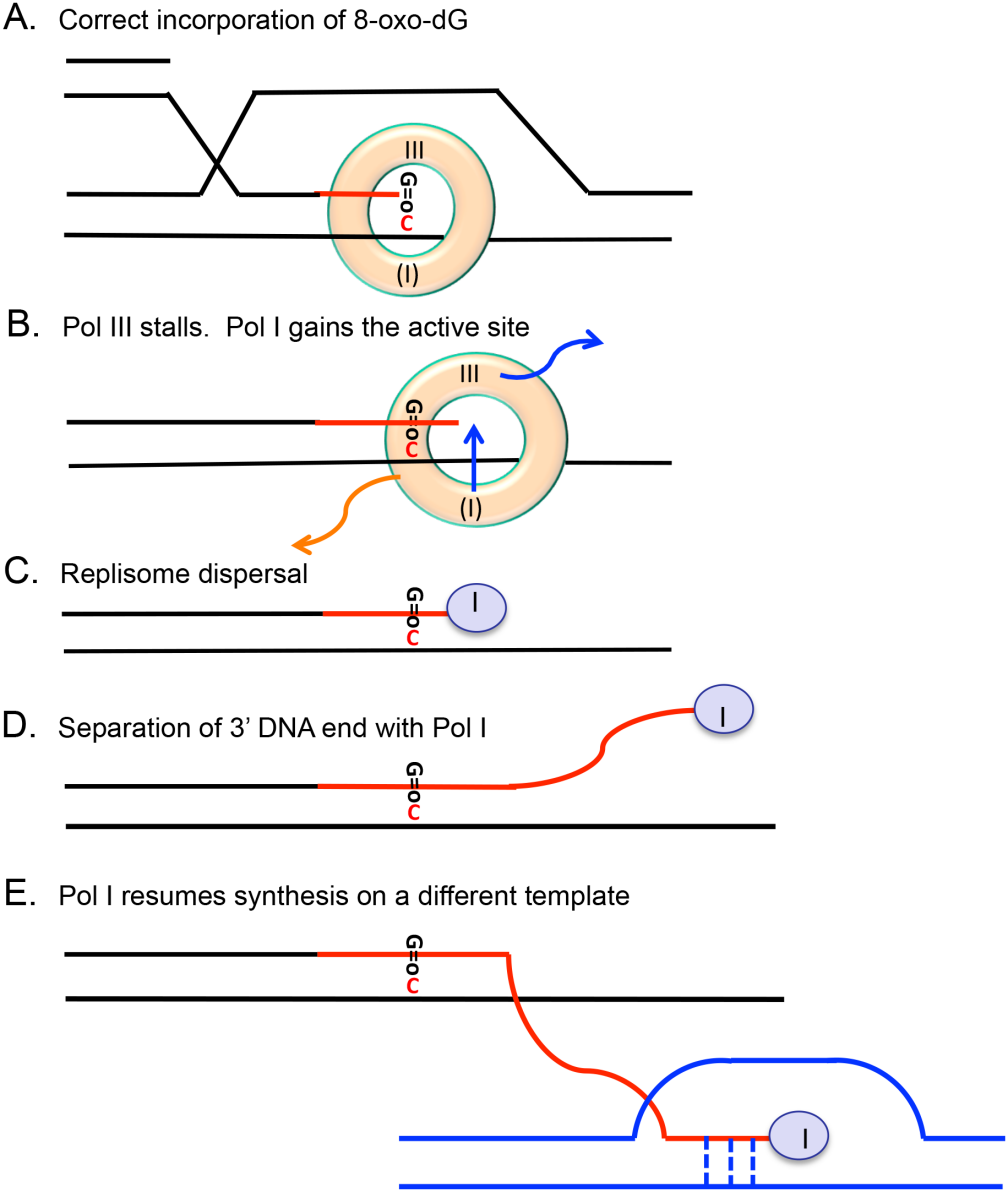
A model for template switching leading to chromosomal rearrangement following incorporation opposite 8-oxo-dG. (**A**) Pausing of the replication complex after Pol III places 8-oxo-dG opposite C [38] (shown, or after Pol III stops after placing C opposite to 8-oxo-G in the template strand, not illustrated) sometimes leads to (**B**) exit of Pol III from active position in the β-clamp, and the DNA polymerase from the inactive position (Pol I here) is switched into the active site. **(C**) Prolonged pausing of the replisome leads to dispersal of the β-clamp. (**D**) The Pol I-bound primer end can become detached, possibly by activation of the editing function, so that template switching can occur, initiating GCR by MMBIR [45, 46]. (**E**) In this case the primer binds to a template at a D-loop at a non-homologous position at microhomology (green) or any other single-stranded DNA (not illustrated). Extension by Pol I will stabilize the junction allowing non-homologous recombination, which creates gene duplications, deletions and other GCRs. Conventions are as in Fig 7 except that the orange arrow indicates detachment of the β-clamp, and the vertical dashed bars in part (E) indicate a microhomologous junction.

Amplification in the Lac assay is mediated by microhomology [45] and is proposed to arise by polymerase template switching during replication by a microhomology-mediated break-induced replication (MMBIR) mechanism [46, 84]. Because Pol I is required for amplification [53], we suggest that Pol I moves to the beta clamp active position when Pol III is stalled (Fig 8B and 8C), and then mediates template switching creating genome rearrangements. We suggest that when Pol I binds the primer end and the replisome is dispersed (Fig 8B, 8C and 8D), as will happen if the fork is stalled for a time [85], Pol I can mediate template switching using very limited homology (Fig 8D and 8E), as has been observed for the Pol I human ortholog DNA PolQ, another A-family polymerase [86–88]. This mechanism (one specific example of which is illustrated in Fig 8), parallels the model for base-substitution and indel formation (one indel version shown in Fig 7). Dissociation of the primer end with Pol I attached might be achieved if the editing function of Pol I is activated by the mismatch at ROS damaged bases, for example at an 8-oxo-dG:C base pair, causing dissociation of the 3'-end to enable it to attain the nuclease domain [89]. Such dissociation would permit polymerase template switching (Fig 8E), the postulated first step in microhomology-mediated recombination.

### Other possible ROS-induced mutagenesis mechanisms

Other possible mechanisms that might have explained the role of ROS in MBR have been described, but do not fit the data presented here. First, because oxidation induces nicks and DSBs directly in DNA [90, 91], and both UV and MMS cause DNA breakage [92, 93], together with the established requirement for DSBs for MBR [6], we considered the possibility that ROS were needed for MBR for the provision of DSBs. We answered this by showing that expression of I-*Sce*I double-strand endonuclease did not raise MBR in a strain over-expressing the SodB superoxide dismutase to the level seen in a strain also expressing I-SceI but not over-expressing SodB (Fig 3). This shows that the loss of MBR associated with reduction in endogenous ROS is not suppressed by provision of DSBs, and therefore that ROS has a role in MBR other than or in addition to DSB formation. Similarly, the action of I-*Sce*I did not suppress the reduction in MBR associated with over-expression of MutT (Fig 5), showing that removal of 8-oxo-dGTP from the deoxynucleotide triphosphate pool is not overcome by provision of DSBs. If ROS were required solely for DSB formation we would have seen MBR at wild-type levels in MutT over-expressing strains.

Second, Pol IV overproduction causes cell death by increasing incorporation of 8-oxo-dG into DNA with subsequent increase in DSBs [54]. This is postulated to be because Pol IV is more likely than replicative polymerases to incorporate 8-oxo-dG, which is then removed by base-excision repair in 8-oxo-dG clusters leading to DSBs [54]. This model cannot account for the role of ROS/8-oxo-dG in MBR because the requirement for 8-oxo-dG was other than or in addition to for generation of DSBs (Figs 3 and 5). Also arguing against the Pol IV/8-oxo-dG-DSB model is the observation that amplification also requires ROS (Fig 2A), and neither SOS-induced levels of Pol IV nor Pol IV itself are required for amplification [21]. Thus the model of Foti et al. [54] does not provide an explanation for the requirement for ROS for MBR.

Third, a model for how single-stranded (ss) DNA damage promotes MBR in yeast also does not explain our data in *E*. *coli*. In yeast, ssDNA regions experience hypermutability both spontaneously and induced by UV light or MMS treatment [94], with mutagenesis increased by orders of magnitude in ssDNA regions at sites of DSB repair by HR, compared with duplex DNA nearby. The ssDNA at sites of HR DSB repair and at uncapped telomeres involved ssDNA lengths longer than 6kb [95]. A similar mechanism is implicated in human cancer cells [12]. This hypermutability in ssDNA is attributed to the absence of complementary DNA sequence during repair of base lesions formed in the ssDNA, causing their replication by mutagenic translesion DNA polymerases [95]. This model appears not to apply to MBR in *E*. *coli* both because the *E*. *coli* MBR mutation clusters cover ~100 kb rather than six [8], and because the model offers no explanation for GCR in MBR.

Finally, ROS were reported previously to participate in an RpoS-independent mechanism of starvation-associated mutation in *E*. *coli* [96]. In contrast with our findings for RpoS-dependent MBR, in that assay, although mutating *mutT*, encoding the 8-oxo-G nucleotide pool sanitizer, increased mutagenesis, mutation of *mutM*, encoding the 8-oxo-dG DNA glycosylase, showed no significant effect, showing no role for 8-oxo-dG in DNA [96, 97], the opposite of our finding (Fig 5). Thus, a different mutagenesis mechanism was at work.

### Roles of ROS and damaged bases in spontaneous mutagenesis

Importantly, the mutagenesis-promoting effect of ROS and 8-oxo-dG in DNA reported here is in *spontaneous* mutation. Although increasing ROS in cells induces mutagenesis (e. g. [25–27]), whether basal levels of spontaneous mutation are also driven by ROS was unknown previously. Previously, the sequence spectrum and frequencies of mutations in anaerobic and aerobic *E*. *coli* cultures suggested that spontaneous base substitutions were oxygen-related, via hydroxyl radicals [98]. The data here show directly that spontaneous SNA and GCR MBR in starving cells require ROS and 8-oxo-dG.

A large body of work has established that organisms respond to stress by increasing their mutation rate under the control of stress responses [13] possibly increasing their ability to evolve and adapt [99]. The demonstration that mutation rate in stressed cells additionally requires base damage caused by ROS, together with our previous finding [57] that there is both positive (by H-NS) and negative (by Dps) regulation of ROS, suggest that cells might regulate their spontaneous mutation rates in response to challenging circumstances by regulating/responding to ROS levels. ROS were long regarded as unwanted byproducts of oxidative metabolism, to be avoided because of the damage that they do to macromolecules. They have been held to be a primary cause of aging [100, 101]. However, it is now apparent that ROS are also an integral component of cell physiology [102]. The use of ROS for accelerated evolution in stressed cells described here might be regarded as a parallel with the involvement of ROS in signaling [103] and in the function of the immune system [104]. Conversely, although it might be advantageous for an organism to mutate specifically when under stress [99, 104, 105], it would be to our advantage to stop pathogens from responding to our immune systems and antibiotics with hypermutation, evasion of the immune system and antibiotic resistance. We have suggested that novel “anti-evolvability” drugs might stop stress-induced evolution of pathogens (and cancers) by suppression of the stress-responses that promote mutagenesis [13].

## Methods

### Construction of *Esherichia coli* K12 strains

The origin of these strains is listed in Table 1. Strains used for Lac^+^ MBR assays are isogenic derivatives of SMR4562, an independent construction of FC40 [106]. Genotypes were confirmed either by PCR or by sequencing as necessary. Mobile plasmid library plasmids [60] containing over-expression alleles of mutants under the control of a P_*tac*_ promoter were conjugated into SMR10866 and SMR10798, strains expressing the chromosomal I-*Sce*I restriction endonuclease controlled by P_*BAD*_, and an I-*Sce*I cutsite, or the enzyme-only control, respectively. Others were constructed by transduction or linear replacement [107] as listed in Table 1. I-*Sce*I cutting was verified by the sensitivity of strains to arabinose that induces I-*Sce*I enzyme.

**Table 1:** *Escherichia coli* K12 trains used in this study.

Strain/Plasmid	Genotype	Source/Reference
CAG12080	*zah281*::Tn*10*	[108]
CH2139	MG1655 *rpsL141*	Christophe Herman
FC36	Δ(*lac proB*)XIII *thi ara* Rif^R^	[42]
FC40	FC36 [F'å45 = F' *proAB*^*+*^ *lacI*^*q*^ *lacI33*Ω*lacZ*]	[42]
PJH18	FC40 [F’ *lac-*amplified]	[43]
PJH33	FC40 [F’ *lac-*amplified]	[43]
PJH51	FC40 [F’ *lac-*amplified]	[43]
PJH1390	SMR4562 Δ*rpoS*::KanFRT	SMR4562xP1(SMR10336)
PJH1399	SMR4562 Δ*rpoS*::FRT	PJH1390 x pCP20
PJH1952	SMR4562 Δ*dps*::KanFRT	SMR4562xP1(JW0797)*
PJH2608	SMR4562 Δ*dps*::FRT	PJH1952 x pCP20
PJH2947	SMR4562 *soxR104 zjc2206*::Tn*10*Kan	SMR4562xP1(JTG1048) [31]
PJH2982	JA200 [pNT3]	[60]
PJH3178	SMR4562 *rpsL141*	SMR4562xP1(CH2139)
PJH3210	JA200 [pNT3/*sodB*]	[60]
PJH3211	JA200[pNT3/*mutM*]	[60]
PJH3231	FC36 Δ*araBAD567* Δ*zie3913*.*1*::tetRtetA+1 FRT Δ*zie3920*.*5*::3ChiKanI-*Sce*Isite Δ*attλ*:: P_*BAD*_I*-Sce*I *dinB50*::FRT [pNT3]	SMR10868 conjugated with [pNT3]
PJH3232	FC36 Δ*araBAD567* Δ*zie3913*.*1*::tetRtetA+1 FRT Δ*zie3920*.*5*::3ChiKanI*Sce*Isite Δ*attλ*:: P_*BAD*_I*Sce*I [pNT3]	SMR10866 conjugated with [pNT3]
PJH3233	FC36 Δ*araBAD567* Δ*zie3913*.*1*::*tetRtetA*+1 FRT Δ*zie3920*.*5*::3ChiKanI-*Sce*Isite Δ*attλ*:: P_*BAD*_I*-Sce*I [pNT3/*mutM*]	SMR10866 conjugated with PJH3211
PJH3237	FC36 Δ*araBAD567* Δ*zie3913*.*1*::tetRtetA+1 FRTcatFRT Δ*attλ*:: P_*BAD*_I*-Sce*I [pNT3]	SMR10798 conjugated with [pNT3]
PJH3240	FC36 Δ*araBAD567* Δ*zie3913*.*1*::tetRtetA+1 FRT Δ*zie3920*.*5*::3ChiKanI-*Sce*Isite Δ*attλ*:: P_*BAD*_I*sce*I *rpoS*::FRT [pNT3]	SMR10865 conjugated with [pNT3]
PJH3255	FC36 Δ*araBAD567* Δ*zie3913*.*1*::*tetRtetA*+1 FRT Δ*zie3920*.*5*::3ChiKanISceIsite Δ*attλ*:: P_*BAD*_I*sce*I [pNT3/*mutY*]	SMR10866 conjugated with PJH3259
PJH3256	FC36 Δ*araBAD567* Δ*zie3913*.*1*::*tetRtetA*+1 FRT Δ*zie3920*.*5*::3ChiKanI-*Sce*Isite Δ*attλ*:: P_*BAD*_I*-Sce*I [pNT3/*mutT*]	SMR10866 conjugated with PJH3258
PJH3257	FC36 Δ*araBAD567* Δ*zie3913*.*1*::*tetRtetA*+1 FRT Δ*zie3920*.*5*::3ChiKanISceIsite Δ*attλ*:: P_*BAD*_I-*sce*I [pNT3/*sodB*]	SMR10866 conjugated with [pNT3/sodB]
PJH3258	JA200 [pNT3/*mutT*]	[60]
PJH3259	JA200 [pNT3/*mutY*]	[60]
PJH3278	SMR4562 *oxyR2 yji-eptC* Kan::FRT	SMR4562xP1(SMR20958)
PJH3295	FC40 *oxyR2 yji-eptC* Kan::FRT *Δdps*::FRT	PJH2608xP1(SMR20958)
SMR601	*ruvC53 eda51*::Tn*10*	[109]
SMR789	FC40 *ruvC53 eda51*::Tn*10*	FC40 x P1(SMR601)
SMR4562	Independent construction of FC40	[106]
SMR4610	SMR4562 *recA*::Tn*10*dCam	[110]
SMR5889	SMR4562 Δ*dinB50*::FRT [F' Δ*dinB50*::FRT]	[41]
SMR6906	SMR4562 *ruvC53 eda*::Tn*10*dCam	By linear replacement [107] into SMR789
SMR8847	SMR4562 [F' *zah281*::Tn*10* Lac^+^]	SMR4562 x P1(CAG12080) [108]
SMR10308	SMR4562 [F' *lafU2*::FRT*cat*FRT *dinB21*(o^c^)]	[41]
SMR10798	FC36 Δ*araBAD567* Δ*zie3913*.*1*::tetRtetA+1 FRT*cat*FRT Δ*attλ*:: P_*BAD*_I-*sce*I	[7]
SMR10865	FC36 Δ*araBAD567* Δ*zie3913*.*1*::tetRtetA+1 FRT Δ*zie3920*.*5*::3ChiKanISceIsite Δ*attλ*:: P_*BAD*_I*sce*I *rpoS*::FRT	[7]
SMR10866	FC36 Δ*araBAD567* Δ*zie3913*.*1*::*tetRtetA*+1 FRT Δ*zie3920*.*5*::3ChiKanI-*Sce*Isite Δ*attλ*:: P_*BAD*_I*sce*I	[7]
SMR10868	FC36 Δ*araBAD567* Δ*zie3913*.*1*::tetRtetA+1 FRT Δ*zie3920*.*5*::3ChiKanI-*Sce*Isite Δ*attλ*:: P_*BAD*_I*-Sce*I *dinB50*::FRT	[7]
SMR12566	SMR4562 Δ*rssB*::*tet*	[75]
SMR15378	SMR4562 Δ*attλ*:: P_*BAD*_*mutLcat*	By replacement of *λ* prophage [111] in SMR8166
SMR20958	MG1655 *Δattλ*::P_*sulA*_*ΩsRBS75mCherry FRT IN-FOG84*	[112]

*All JW strains are from the Keio Collection, described in [113].

### Lac^+^ MBR assays

Experimental procedures are as described [53]. The strains to be compared are grown at 37° for two days in M9 medium with 0.1% glycerol and thiamine, then mixed with a 25-fold excess of Δ*lac* scavenger cells, and plated in top agar on M9 minimal lactose and thiamine solid medium. Plates are incubated at 37°, and Lac^+^ revertant colonies counted daily. About half of the colonies appearing on day 2 result from Lac^+^ mutant cells that arose during growth. Stress-induced mutant colonies appear linearly from day 3 onward. These experiments were continued to day 7 to obtain measurements of *lac-*amplification, which forms colonies later than SNA mutations [43]. The mutation rate (mutants per cell per day) is taken from the linear part of the curve as the mean and SEM of three or four parallel cultures of each strain. To determine cell viability during the prolonged starvation, the Lac^-^ lawn is sampled at intervals by plating cells on complete medium containing rifampicin, which does not allow growth of the scavenger cells. 5-bromo-4-chloro-3-indolyl-β-D-galactopyranoside (x-gal) is included in the medium so that only Lac^-^ cells are counted. In all experiments reported, Lac^-^ viable cell counts did not vary significantly over the course of the experiment. Bipyridine and TU were added to the solid lactose minimal medium on which the starved cells were spread. Bipyridine was dissolved in ethanol. TU was dissolved in distilled water. Both solutions were filter-sterilized. Errors on all reported experiments are one standard error of the mean (SEM) of at least three independent experiments of three or four cultures per strain per experiment. To distinguish indel from amplified Lac^+^ cfu, samples of Lac^+^ colonies were replated onto rich medium containing x-gal dye on which SNAs produce solid blue colonies and amplified isolates produce sectored blue and white colonies [43]. MutL, expressed from the chromosome regulated by a P_*BAD*_ promoter, was derepressed by absence of glucose in the medium during growth and on the minimal medium lactose plates.

### Chromosomal Tet MBR assays

Experimental procedures are described in [7]. Four cultures of each strain are grown in M9 glycerol liquid medium with 20μg/ml carbenicillin for plasmid maintenance, 50μg/ml proline and with 0.1% glucose to repress P_*BAD*_, back-diluted after 12 hours twice and cultured for 84 hours. I-*Sce*I endonuclease is induced by exhaustion of the glucose in the medium. Samples are then plated on complete medium with 0.1% glucose with and without tetracycline to obtain the Tet^R^ mutant frequency, and colonies are counted after one day. Activity of I-*Sce*I was confirmed for all cultures during the experiments by their inability to grow with 0.0001% arabinose in M9 glycerol medium with IPTG (S2A Fig). I-*Sce*I cutting was confirmed for each strain by the loss of viability when plated on medium without glucose containing 0.001% arabinose (S2B Fig). Error bars on all reported data are the SEM of at least 3 independent experiments. Genes carried in mobile plasmid library plasmids [60] were introduced to strains containing inducible DSB I-*Sce*I cut-sites and enzyme and induced by adding 1mM IPTG to the liquid cultures during growth. A control strain containing an empty overexpression plasmid vector and an I-*Sce*I cutsite and I-SceI enzyme cassette was also similarly treated with IPTG and plated to control for effects of plasmid expression on DSB induction and growth.

### Ultraviolet irradiation and MMS treatment

Immediately prior to plating, aliquots of Lac-assay cells in prolonged stationary phase per the Lac MBR assay, were exposed to either UV-C irradiation or MMS. For MMS treatment, 1 mL of starved cell culture was pulse treated with MMS at 5 or 10mM, freshly diluted in water, for 20 minutes at 37°C. For UV-C irradiation, 1 mL aliquots of each culture were irradiated at either 5 J/m^2^, or 10 J/m^2^ using a Stratalinker 2400 UV lamp emitting at 254nm. Viable cell count was determined after MMS and UV treatment to confirm that there was no loss of viability.

## Supporting information

S1 Fig Bipyridine and TU do not affect cell viability or time to colony formation under MBR assay conditions. Reconstruction experiments, using SMR4562, in which a Lac^+^ indel revertant and three *lac-*amplified strains were mixed with Δ*lac* scavenger cells and plated in precise reconstructions of mutant selection conditions show that neither treatment with TU nor 2'2-bipyridine reduces **(A)** cell viability or **(B)** the speed of formation of Lac^+^ revertant colonies under experimental assay conditions. The data indicate that reductions in yields of Lac^+^ colonies in MBR experiments with TU or bip treatment reflect reduction of mutagenesis, not inability of mutant cells to form colonies in the presence of those ROS-reducing agents. Left panels, bip treatment; right panels, TU treatment.
(DOCX)

S2 Fig Induction of mobile over-expression plasmids with IPTG does not block formation of double-strand breaks by P_*BAD*_-regulated I-*Sce*I endonuclease. **(A).** Activity of I-*Sce*I was confirmed for all cultures during the experiments by their inability to grow with 0.0001% arabinose in M9 glycerol medium with IPTG (arabinose medium). Only cultures of strains lacking the I-*Sce*I cutsite showed significant growth. **(B).** I-*Sce*I cutting in the presence of induced mobile plasmids genes was confirmed by their loss of viability at higher arabinose concentrations. Chromosomal P_*BAD*_-I*Sce*I cassettes were induced with 0.001% arabinose in the presence and absence of 1 mM IPTG and DSB formation via I-*Sce*I cleavage was measured as the frequency of arabinose-sensitive cfu among total viable cells (assayed on glucose). Student’s *t*-tests found no significant differences between the frequencies of arabinose-resistance in the presence or absence of IPTG in strains expressing vector only (PJH3232, *p* = 0.32), pSodB (PJH3257, *p* = 0.77), pMutT (PJH3256, *p* = 0.67), and pMutM (PJH3233, *p* = 0.60) mobile plasmids. Frequencies of arabinose-resistance were taken for three cultures per strain per experiment and frequencies shown are the means of 3 experiments with error bars representing ± SEM. Cells were taken from cultures 52 hours into the Tet assay protocol and plated on M9 minimal medium with 0.1% glucose for viable cell titer, and on M9 minimal medium containing 0.1% glycerol and 0.001% arabinose to induce I-*Sce*I. Other supplements were as described in Methods.
(DOCX)

S3 Fig Induction of *mutM*, *mutT*, *mutY* and *sodB* do not delay growth of cell cultures. Examples from two different experiments. Three 3ml cultures were grown overnight in M9 glucose medium with proline and carbenicillin at 37°, and diluted 100-fold in the same medium with or without IPTG. 200ml of each culture was inoculated in triplicate into each medium, randomized in 96-well plates and change in OD 600 was monitored for 24 h. Most strains show some increase in growth upon induction by IPTG and none shows inhibition. Strains used were those employed in the Tet assay mutation experiments.
(DOCX)
