# Supplementary material for: Persistent damaged bases in DNA allow mutagenic break repair in Escherichia coli
Source: PLoS Genet. 2017 Jul 20;13(7):e1006733. doi: 10.1371/journal.pgen.1006733 (PMC5542668; doi:10.1371/journal.pgen.1006733)

**Fig. S1. Bipyridine and TU do not affect cell viability or time to colony formation under MBR assay conditions.** Reconstruction experiments, using SMR4562, in which a Lac^+^ indel revertant and three *lac-*amplified strains were mixed with ∆*lac* scavenger cells and plated in precise reconstructions of mutant selection conditions show that neither treatment with TU nor 2'2-bipyridine reduces **(A)** cell viability or **(B)** the speed of formation of Lac^+^ revertant colonies under experimental assay conditions. The data indicate that reductions in yields of Lac^+^ colonies in MBR experiments with TU or bip treatment reflect reduction of mutagenesis, not inability of mutant cells to form colonies in the presence of those ROS-reducing agents. Left panels, bip treatment; right panels, TU treatment.


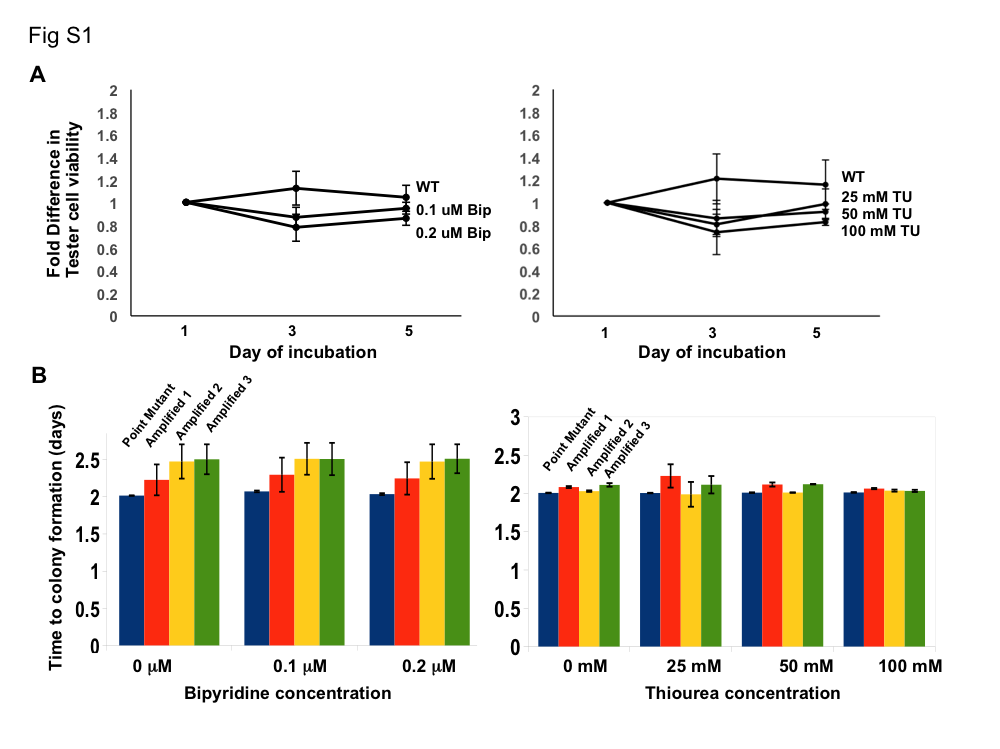

Supplement: S1 Fig — Reconstruction experiments, using SMR4562, in which a Lac+ indel revertant and three lac-amplified strains were mixed with Δlac scavenger cells and plated in precise reconstructions of mutant selection conditions show that neither treatment with TU nor 2'2-bipyridine reduces (A) cell viability or (B) the speed of formation of Lac+ revertant colonies under experimental assay conditions. The data indicate that reductions in yields of Lac+ colonies in MBR experiments with TU or bip treatment reflect reduction of mutagenesis, not inability of mutant cells to form colonies in the presence of those ROS-reducing agents. Left panels, bip treatment; right panels, TU treatment. (DOCX) [file pgen.1006733.s001.docx]
