# Supplementary material for: Persistent damaged bases in DNA allow mutagenic break repair in Escherichia coli
Source: PLoS Genet. 2017 Jul 20;13(7):e1006733. doi: 10.1371/journal.pgen.1006733 (PMC5542668; doi:10.1371/journal.pgen.1006733)

**Fig. S2. Induction of mobile over-expression plasmids with IPTG does not block formation of double-strand breaks by** P*_BAD_***-regulated I-*Sce*I endonuclease.**

**(A).** Activity of I-*Sce*I was confirmed for all cultures during the experiments by their inability to grow with 0.0001% arabinose in M9 glycerol medium with IPTG (arabinose medium). Only cultures of strains lacking the I-*Sce*I cutsite showed significant growth. **(B).** I-*Sce*I cutting in the presence of induced mobile plasmids genes was confirmed by their loss of viability at higher arabinose concentrations. Chromosomal P*_BAD_*-I*Sce*I cassettes were induced with 0.001% arabinose in the presence and absence of 1 mM IPTG and DSB formation via I-*Sce*I cleavage was measured as the frequency of arabinose-sensitive cfu among total viable cells (assayed on glucose). Student’s *t*-tests found no significant differences between the frequencies of arabinose-resistance in the presence or absence of IPTG in strains expressing vector only (PJH3232, *p* = 0.32), pSodB (PJH3257, *p* = 0.77), pMutT (PJH3256, *p* = 0.67), and pMutM (PJH3233, *p* = 0.60) mobile plasmids. Frequencies of arabinose-resistance were taken for three cultures per strain per experiment and frequencies shown are the means of 3 experiments with error bars representing ± SEM. Cells were taken from cultures 52 hours into the Tet assay protocol and plated on M9 minimal medium with 0.1% glucose for viable cell titer, and on M9 minimal medium containing 0.1% glycerol and 0.001% arabinose to induce I-*Sce*I. Other supplements were as described in Methods.


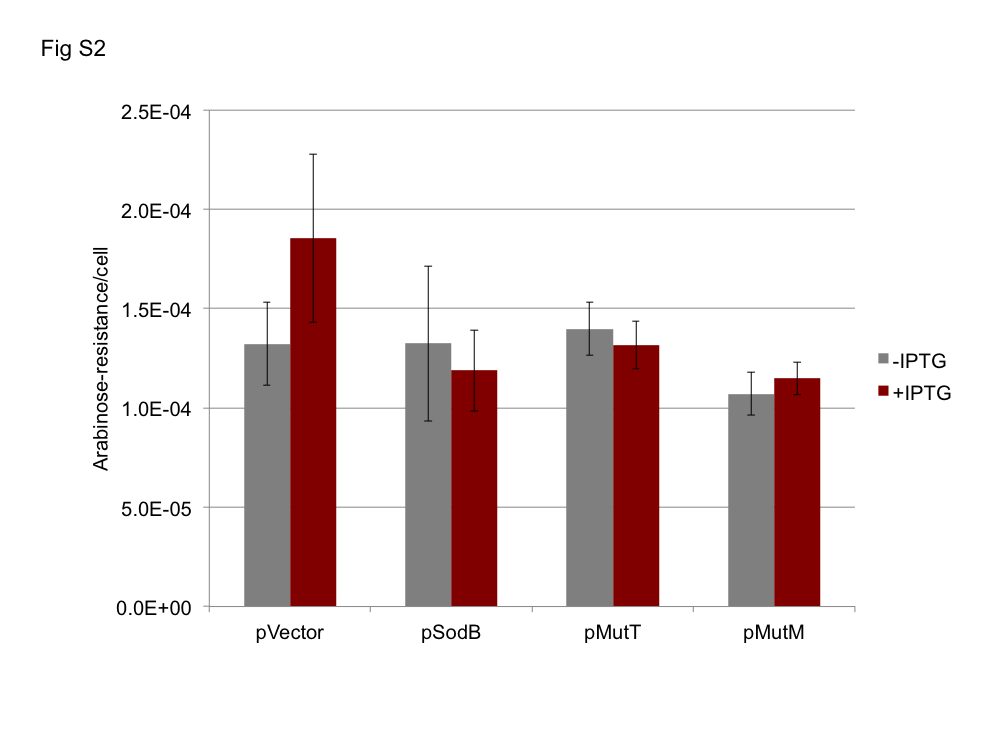

Supplement: S2 Fig — (A). Activity of I-SceI was confirmed for all cultures during the experiments by their inability to grow with 0.0001% arabinose in M9 glycerol medium with IPTG (arabinose medium). Only cultures of strains lacking the I-SceI cutsite showed significant growth. (B). I-SceI cutting in the presence of induced mobile plasmids genes was confirmed by their loss of viability at higher arabinose concentrations. Chromosomal PBAD-ISceI cassettes were induced with 0.001% arabinose in the presence and absence of 1 mM IPTG and DSB formation via I-SceI cleavage was measured as the frequency of arabinose-sensitive cfu among total viable cells (assayed on glucose). Student’s t-tests found no significant differences between the frequencies of arabinose-resistance in the presence or absence of IPTG in strains expressing vector only (PJH3232, p = 0.32), pSodB (PJH3257, p = 0.77), pMutT (PJH3256, p = 0.67), and pMutM (PJH3233, p = 0.60) mobile plasmids. Frequencies of arabinose-resistance were taken for three cultures per strain per experiment and frequencies shown are the means of 3 experiments with error bars representing ± SEM. Cells were taken from cultures 52 hours into the Tet assay protocol and plated on M9 minimal medium with 0.1% glucose for viable cell titer, and on M9 minimal medium containing 0.1% glycerol and 0.001% arabinose to induce I-SceI. Other supplements were as described in Methods. (DOCX) [file pgen.1006733.s002.docx]
