# Supplementary material for: Persistent damaged bases in DNA allow mutagenic break repair in Escherichia coli
Source: PLoS Genet. 2017 Jul 20;13(7):e1006733. doi: 10.1371/journal.pgen.1006733 (PMC5542668; doi:10.1371/journal.pgen.1006733)

**Fig. S3. Induction of *mutM, mutT, mutY* and *sodB* do not delay growth of cell cultures.** Examples from two different experiments. Three 3ml cultures were grown overnight in M9 glucose medium with proline and carbenicillin at 37°, and diluted 100-fold in the same medium with or without IPTG. 200ml of each culture was inoculated in triplicate into each medium, randomized in 96-well plates and change in OD 600 was monitored for 24 h. Most strains show some increase in growth upon induction by IPTG and none shows inhibition. Strains used were those employed in the Tet assay mutation experiments.


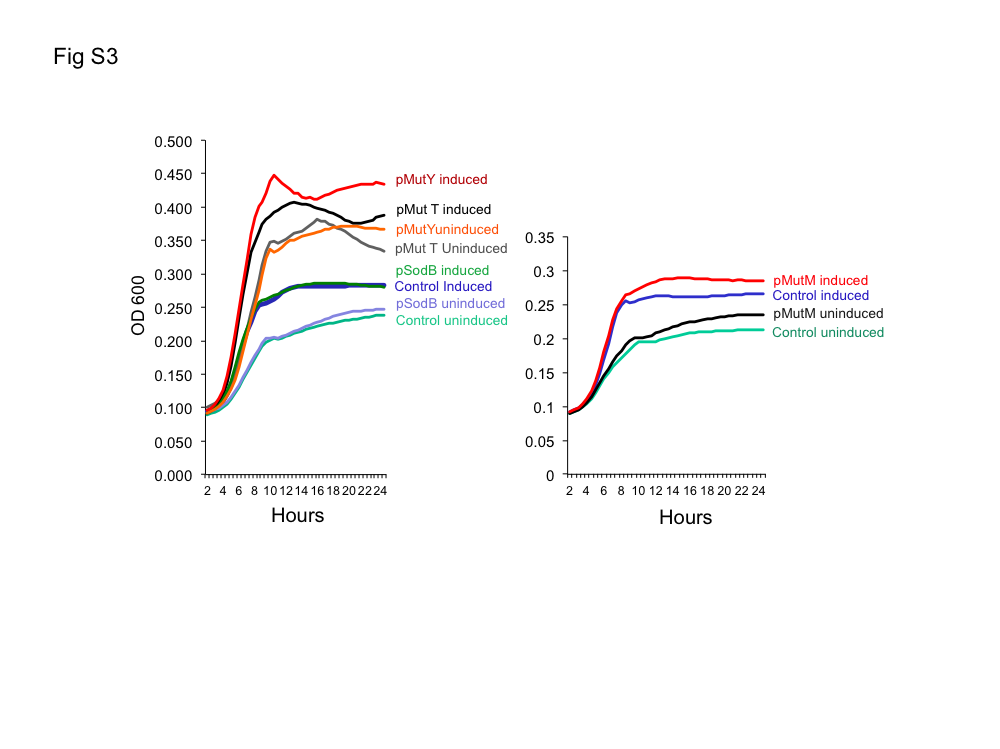

Supplement: S3 Fig — Examples from two different experiments. Three 3ml cultures were grown overnight in M9 glucose medium with proline and carbenicillin at 37°, and diluted 100-fold in the same medium with or without IPTG. 200ml of each culture was inoculated in triplicate into each medium, randomized in 96-well plates and change in OD 600 was monitored for 24 h. Most strains show some increase in growth upon induction by IPTG and none shows inhibition. Strains used were those employed in the Tet assay mutation experiments. (DOCX) [file pgen.1006733.s003.docx]
